# Catmint (*Nepeta nuda* L.) Phylogenetics and Metabolic Responses in Variable Growth Conditions

**DOI:** 10.3389/fpls.2022.866777

**Published:** 2022-05-16

**Authors:** Detelina Petrova, Uroš Gašić, Lyubomira Yocheva, Anton Hinkov, Zhenya Yordanova, Ganka Chaneva, Desislava Mantovska, Momchil Paunov, Lyubomira Ivanova, Mariya Rogova, Kalina Shishkova, Daniel Todorov, Anita Tosheva, Veneta Kapchina-Toteva, Valya Vassileva, Atanas Atanassov, Danijela Mišić, Georgi Bonchev, Miroslava Zhiponova

**Affiliations:** ^1^Department of Plant Physiology, Faculty of Biology, Sofia University “St. Kliment Ohridski”, Sofia, Bulgaria; ^2^Department of Plant Physiology, Institute for Biological Research “Siniša Stanković”—National Institute of the Republic of Serbia, University of Belgrade, Belgrade, Serbia; ^3^Department of Biology, Medical Genetics and Microbiology, Faculty of Medicine, Sofia University “St. Kliment Ohridski”, Sofia, Bulgaria; ^4^Laboratory of Virology, Faculty of Biology, Sofia University “St. Kliment Ohridski”, Sofia, Bulgaria; ^5^Department of Biophysics and Radiobiology, Faculty of Biology, Sofia University “St. Kliment Ohridski”, Sofia, Bulgaria; ^6^Department of Botany, Faculty of Biology, Sofia University “St. Kliment Ohridski”, Sofia, Bulgaria; ^7^Department of Molecular Biology and Genetics, Institute of Plant Physiology and Genetics, Bulgarian Academy of Sciences, Sofia, Bulgaria; ^8^Joint Genomic Center, Sofia, Bulgaria

**Keywords:** antibacterial, antioxidant, antiviral, DNA barcoding, iridoids, light, *Nepeta nuda*, phenolics

## Abstract

*Nepeta nuda* (catmint; Lamiaceae) is a perennial medicinal plant with a wide geographic distribution in Europe and Asia. This study first characterized the taxonomic position of *N. nuda* using DNA barcoding technology. Since medicinal plants are rich in secondary metabolites contributing to their adaptive immune response, we explored the *N. nuda* metabolic adjustment operating under variable environments. Through comparative analysis of wild-grown and *in vitro* cultivated plants, we assessed the change in phenolic and iridoid compounds, and the associated immune activities. The wild-grown plants from different Bulgarian locations contained variable amounts of phenolic compounds manifested by a general increase in flowers, as compared to leaves, while a strong reduction was observed in the *in vitro* plants. A similar trend was noted for the antioxidant and anti-herpesvirus activity of the extracts. The antimicrobial potential, however, was very similar, regardless the growth conditions. Analysis of the *N. nuda* extracts led to identification of 63 compounds including phenolic acids and derivatives, flavonoids, and iridoids. Quantification of the content of 21 target compounds indicated their general reduction in the extracts from *in vitro* plants, and only the ferulic acid (FA) was specifically increased. Cultivation of *in vitro* plants under different light quality and intensity indicated that these variable light conditions altered the content of bioactive compounds, such as aesculin, FA, rosmarinic acid, cirsimaritin, naringenin, rutin, isoquercetin, epideoxyloganic acid, chlorogenic acid. Thus, this study generated novel information on the regulation of *N. nuda* productivity using light and other cultivation conditions, which could be exploited for biotechnological purposes.

## Introduction

The mint family Lamiaceae has been well known for its importance as a source of aromatic oil, wood, ornamentals, culinary and medicinal herbs, which determines the strong interest toward the detailed studies of related members (Mint Evolutionary Genomics Consortium, 2018). Lamiaceae has been annotated as the sixth largest angiosperm family including 12 subfamilies (Zhao et al., 2021). The subfamily Nepetoideae is monophyletic and comprises nearly all aromatic species within Lamiaceae and the presence of rosmarinic acid (RA) is one of their characteristics (Harley et al., 2004). Nepetoideae is the largest subfamily (~3,400 species) spread throughout all continents except Antarctica (Hedge, 1990; Harley et al., 2004), which is divided into three tribes, Elsholtzieae, Mentheae, and Ocimeae (Harley et al., 2004). The tribe Mentheae comprises the largest number of genera and species of any tribe within Nepetoideae and Lamiaceae (Zhao et al., 2021). Many plants in this group are of great economic and ecological importance, which attracts the attention of scientists (Zhao et al., 2021).

The genus *Nepeta* L. (Catmints) includes species that are mostly herbaceous perennials (Formisano et al., 2011; Süntar et al., 2017; Salehi et al., 2018; Aćimović et al., 2020; Sharma et al., 2021). In the Bulgarian flora, the genus *Nepeta* is represented by 4 species: *Nepeta cataria* L., *N. nuda* L., *N. parviflora* M. Bieb. and *N. ucrainica* L. (Assyov et al., 2012). *Nepeta nuda* L. belongs to the Euro-Asiatic floristic element and it is widely distributed across Bulgaria in all floristic regions and over a wide range of elevations up to 1900 m a.s.l. (Assyov et al., 2012). *Nepeta nuda* can grow at full sun to light shade conditions, usually in woodlands, meadows, and fencerows (Pádure, 2004). A previous report placed *N. nuda* among the most frequently visited by honeybees melliferous plants (Bozek, 2003). In Bulgaria, *N. nuda* is known also as “naked (or hairless) catmint” that likely refers the naked or sparse short hairy stem and leaves (Asenov, 1989).

It has been shown that *N. nuda* differs from the most studied *N. cataria* (catnip) by the lack or decreased amounts of monoterpenoid nepetalactones (Baser et al., 2000; Mišić et al., 2015). Nepetalactones are the major chemical constituents of the essential oil in many *Nepeta* species. They can be contained in large amounts, occur either in low amounts or absent entirely (Baser et al., 2000). Three different *Nepeta* chemotypes have been recognized as follows: (1) Nepetalactone, (2) caryophyllene oxide, and (3) 1,8-cineole/linalool. According to previous studies, *N. nuda* belongs to the 1,8-cineole chemotype although the composition of the essential oil could depend on the environmental conditions, plant growth stage, and analytical method (De Pooter et al., 1987; Handjieva et al., 1996; Chalchat et al., 1998; Kökdil et al., 1998; Pádure et al., 2008; Alim et al., 2009; Gkinis et al., 2010; Kilic et al., 2011; Bozari et al., 2013; Mišić et al., 2015; Mamadalieva et al., 2016; Baranauskiene et al., 2019). Nepetalactones have both insect pheromone and insect repellent activities against the phytophagous insects (Eisner, 1964; Uenoyama et al., 2021). Catmint nepetalactones are known to excise domestic cats and many wild felid species including lions, tigers, and ocelots, however, the benefit for the mint plants is still unclear (Eisner, 1964; Lichman et al., 2020). In the traditional medicine, the *N. nuda* decoction is used internally against cystitis prostate gland inflammation, externally against wounds, and on the stock udder for mastitis treatment (Kozhuharova et al., 2014). The genus *Nepeta* is rich in iridoid glycosides and phenolic compounds that exhibit a broad spectrum of pharmacological properties, such as antioxidant, antimicrobial, phytotoxic, antiparasitic, and antiviral activities (Handjieva et al., 1996; Formisano et al., 2011; Süntar et al., 2017; Sharma et al., 2021). A recent study demonstrates that two Balkan endemics, *N. rtanjensis* Diklić & Milojević and *N. argolica* Bory & Chaub. in Bory subsp. *argolica*, contain two nepetalactones and 1,5,9-epideoxyloganic acid (1,5,9-*e*DLA) as major iridoids (Aničić et al., 2021). The composition and importance of *N. nuda* phenolic compounds have been also previously reported, giving a special emphasis to phenolic acids, their derivatives, and flavonoids (Mišić et al., 2015; Aras et al., 2016b; Fraga et al., 2017; Dienaite et al., 2018; Smiljković et al., 2018; Sarikurkcu et al., 2019; Hinkov et al., 2020; Aničić et al., 2021).

The secondary metabolites can play a main role in phytoimmuniy acting as phytotoxins for competing plants, pathogens, and herbivorous organisms (Mithöfer and Boland, 2012; Cheng and Cheng, 2015). The content of phenolic compounds can correlate with the bioactivity of *N. nuda* extracts. Aqueous extracts exhibit allelopathic activity by inhibiting the growth of wheat and cucumber (Dragoeva et al., 2017). On the other hand, they possess antioxidant, anti-proliferative and antiviral activities (Dienaite et al., 2018; Hinkov et al., 2020). *Nepeta nuda* tincture is effective against oral pathogens (Smiljković et al., 2018). A study on the antiparasitic activity showed a very high potential of methanol and hexane extracts of *N. nuda* against the parasite *Tripanozoma brucei rhodesiense* without having toxic side effects on the host cells (Kirmizibekmez et al., 2011). Environmental factors such as light, temperature (Alberti, 2020; Zhiponova et al., 2020a), and soil composition (Pádure et al., 2008; Zhiponova et al., 2020b), additionally affect plant growth and biosynthetic potential. Compared to wild-grown plants, the *in vitro* cultivation ensures controlled and sterile conditions on a nutrient medium of known composition. The main disadvantage of *in vitro* cultivation is the slow growth and altered metabolic activity of the plant (Kapchina-Toteva et al., 2014; Yordanova et al., 2017). *Nepeta nuda* has been propagated *in vitro* and successfully adapted to *ex vitro* conditions (Nedelkova et al., 2012; Dragolova et al., 2015).

The first aim of this work was to establish DNA barcode regions for precise taxonomic identification of *N. nuda*. Next, we investigated the environmental impact on *N. nuda* immune response including metabolic adjustment. The interdependence of various biological activities (antioxidant, antiviral, and antibacterial), as linked to phenolic and iridoid compounds in flowers and leaves of wild-grown vs. *in vitro* cultivated *N. nuda* was assessed. Since the optimal bioactive potential of wild-grown plants occurs during flower development, in the proposed *in vitro* cultivation system a light formula stimulating flowering by different light intensities was tested. The effect of light composition and intensity was estimated regarding phytochemical composition.

## Materials and Methods

### Plant Material

*Nepeta nuda* plants were collected from natural habitats (Pirin; Rhodopes; and two samples Rila #1 and Rila #2 collected from the same location with 3-week difference) in Bulgaria during the flowering period (Supplementary Table S1). Samples of vouchers were deposited to the Herbarium of Sofia University “St. Kliment Ohridski,” Sofia, Bulgaria (the vouchers are listed in Supplementary Table S1). *In vitro* plants were maintained in the collection of the Department of Plant Physiology at the Faculty of Biology, as described by Nedelkova et al. (2012) and Dragolova et al. (2015). The plants were grown under sterile controlled conditions on MS (Murashige and Skoog, 1962) medium with 3% sucrose and 0.7% agar under white light (80 μmol m^−2^s^−1^ photosynthetic active radiation, cool white fluorescent TL-D 36W/54-765 1SL/25 Philips), photoperiod 16 h light/8 h dark, 25±1°C, 60–70% humidity. The plants were passaged every 4 weeks *via* shoot tips. The *ex vitro* plants were adapted for 3 months in a greenhouse and transferred to soil for 1 year. Wild-grown *N. nuda* (*in situ* Pirin and *ex vitro*) and *in vitro* cultured plants (Dragolova et al., 2015) were used for comparative analyses (*n* ≥ 15; Supplementary Table S1). For dry weight (DW), the plants were dried in the dark at room temperature. The plant extracts preparation is described in Supplementary Material and summarized in Supplementary Table S2.

For the light treatments, the internodes from *in vitro* cultivated plants were grown for 5 weeks under different light quality and intensity as discussed in the following: White (W) light (80 μmol m^−2^ s^−1^; fluorescent) as a control, and a combination of blue and red light (blue:red:far red 15%:75%:10%) with two different light intensities: BR (240 μmol m^−2^ s^−1^; LED), and BRS (40 μmol m^−2^ s^−1^, LED, i.e., as “shadowed”) (Zhiponova et al., 2020a). The similar-looking plants were selected for analysis (*n* ≥ 20).

### The DNA Barcoding Analysis

#### Genomic DNA Isolation, PCR, and Sequencing

The total genomic DNA was isolated using the DNeasy Plant Kit (QIAGEN) from frozen leaf material from *in vitro N. nuda* plants (Supplementary Table S1). The DNA barcode sequences of four different DNA regions are as follows: In the nuclear (ITS) (nuclear ribosomal internal transcribed spacer), *rbcL* (plastid gene encoding *ribulose-1,5-bisphosphate carboxylase/oxygenase large subunit*), *matK* (plastid gene encoding *maturase K, tRNA-Lys*), and *trnH-psbA* (plastid intergenic spacer region) were analyzed. The primer sequences (synthesized by Microsynth, Gottingen, Germany) and PCR conditions are shown in Supplementary Table S3. PCR amplification was performed in 20-μl reaction mixtures containing approximately 30 ng of genomic DNA, 1 × PCR buffer, MgCl^2^ (2.0 mM for ITS, 1.5 mM for *matK*, 2.5 mM for *rbcL*, and *trnH-psbA*), 0.2 mM of each dNTP, 0.2 μM of each primer, and 1.0 U Taq DNA Polymerase (Solis BioDyne). The quality of PCR products was checked on 1% agarose gel in TBE buffer containing GoodViewTM staining dye. Successful amplicon products were sequenced in both directions by Microsynth with the primers used for PCR amplification (Supplementary Table S4).

#### Sequence Analyses and Construction of Phylogenetic Trees

The DNA sequences of *in vitro N. nuda* for each barcode region (Supplementary Table S4) were edited in Molecular Evolutionary Genetics Analysis (MEGA) ver. 10.2.4 software (Kumar et al., 2018). The Basic Local Alignment Search Tool (BLAST) implemented in MEGA and the Barcode of Life Data System (BOLD) database (Ratnasingham and Hebert, 2007) were used to retreive sequences similar to *N. nuda*. The set of database sequences and respective DNA barcodes for *N. nuda* were subjected to CLUSTAL W alignment in MEGA. The alignment files were used for the construction of phylogenetic trees in the software program Geneious (Biomatters, New Zealand) using the genetic distance Tamura–Nei model (Tamura and Nei, 1993), the clustering method UPGMA and the resampling method bootstrap with 500 replicates. The alignment files for genes *rbcL, matK*, and *trnH-psbA* were concatenated by name in Geneious software after making database accession names unified to species level. The derived concatenated file was used to obtain a consensus tree applying the same parameters as for individual DNA barcode trees.

### Quantification of Total Phenolics and Total Flavonoids Content

The procedures for spectrophotometric quantification of phenolics and flavonoids are described by Zhiponova et al. (2020b), and performed with spectrophotometer Shimadzu UV 1800 (Kyoto, Japan). The content of polyphenols was determined according to Singleton et al. (1999) by a standard curve created using the known concentrations of gallic acid (GA). The content of flavonoids was measured according to the protocol of Chang et al. (2002) by a standard curve based on the known concentrations of quercetin (Q).

### Determination of Antioxidant Activity

The extracts obtained by different solvents (Supplementary Table S2A) were tested to determine 50% effective concentration (EC_50_) for radical scavenging activity using stable 2,2'-diphenyl-1-picrylhydrazyl (DPPH) free radical (Blois, 1958). The extract concentrations between 50 and 200 μg ml^−1^ were prepared in 1.5 ml methanol followed by the addition of 0.5 ml of 0.1 mM DPPH. Methanol was used as a blank and a mixture of methanol (1.5 ml) and DPPH (0.5 ml) was utilized as a negative control. The samples were incubated for 30 min in the dark, and the absorbance was measured at λ = 517 nm with spectrophotometer Shimadzu UV 1800 (Kyoto, Japan). The results are given as EC_50_ in μg ml^−1^ extract.

The crude methanol extracts from plant variants were prepared as described in Supplementary Material and presented in Supplementary Table S2B. The DPPH-radical-scavenging activity was measured using Trolox as a standard, according to the method of Brand-Williams et al. (1995). The reaction included 80 μl extract (1 mg DW ml^−1^) and 1920 μl 0.06 mM DPPH solution in methanol, and the samples were incubated at room temperature in the dark for 30 min. After the calibration against methanol, the absorbance was detected at λ = 515 nm with spectrophotometer Shimadzu UV 1800 (Kyoto, Japan). The radical–scavanging activity was measured in μM Trolox ml^−1^ *via* a standard curve. The DPPH–scavanging activity was expressed as mM Trolox per gram DW (mM Trolox gDW^−1^).

### Antiviral Activity

Madine and Darby bovine kidney (MDBK) cells (purchased from ATCC) were used and grown in Dulbecco's Modified Eagle Medium (DMEM, Sigma–Aldrich) supplemented with 8% fetal calf serum (FCS, Sigma–Aldrich) (with 8 μg ml^−1^ gentamycin, SANDOZ, and 10 mM HEPES buffer, Sigma–Aldrich). The maintenance medium contained FCS with reduced concentration of 4%. The strain F of *Human alphaherpesvirus* type 1 (HHV-1) (purchased from ATCC) was propagated in MDBK cells and stored at −70°C (BINDER GmbH, Germany) until used. This HHV-1 strain is sensitive to the antiviral agent acyclovir (Hinkov et al., 2020). The virus titer was determined by cytopathic effect (CPE) assay using the method of Reed and Muench (1938) and plaque assay (Dulbecco, 1952) in MDBK.

#### Cytotoxicity Assay

The cell toxicity was monitored by determining the effect of the *N. nuda* extracts on MDBK cell viability by a colorimetric method (MTT assay) (Mosmann, 1983). The confluent monolayers of MDBK cells in 96-well plates (Corning®) were overlaid with 0.1 ml/well maintenance medium, 0.1 ml/well of the serial 2-fold dilutions of the extracts (3 well/dilution), and incubated at 37°C (BINDER GmbH, Germany) for 72 h. At least three wells in the plate were used as controls without a treatment (0.1 ml/well maintenance medium added only). On the third day, 20 μl of MTT (Sigma–Aldrich) [5 mg ml^−1^ in phosphate buffered saline (PBS, Sigma–Aldrich)] was added to each cell; the monolayers were incubated for 2 h at 37°C. The resulting formazan precipitate was dissolved in dimethyl sulfoxide (DMSO). After the incubation for few minutes at room temperature to ensure that all crystals were dissolved, the absorbances at λ = 540 nm were measured (ELISA reader Multiscan MX, ThermoFisher Scientific, USA). The percentage of viable treated cells was calculated relative to the untreated controls: [(OD)exp.)/(ODcell control)] × 100, where (ODexp.) and (ODcell control) indicate the absorbance of the test sample and the cell control, respectively. The maximum non-toxic concentration (MNC) was defined as the highest extract concentration that did not cause damage or death to the treated MDBK cells. The 50% cytotoxicity concentration (CC_50_) was determined as the test compound concentration required for the reduction of cell viability by 50%.

#### The MTT-Based Colorimetric Assay for Detection of HHV Replication Inhibition

A modification of the MTT assay developed for screening anti-HHV compounds (Takeuchi et al., 1991) was used. Two experimental setups differing in the time of extract addition were employed. The confluent monolayers in 96-well plates were overlaid with 0.1 ml/well of virus suspension (low multiplicity of infection (MOI) 100 TCID 50 (tissue culture infectious dose 50)/well). In the first experimental setup, 0.1 ml serial 2-fold dilutions of the extracts (at least 3 wells/dilution) or 0.1 ml maintenance medium were added into wells. The latter served as a virus control immediately after the inoculation. In the second experimental setup, the plates were incubated for 1 h at 37°C to allow the virus to adsorb and dilutions of the extracts or maintenance medium (control) were subsequently added. Uninoculated cells were used as a cell control. On the fifth day, 20 μl of MTT (5 mg ml^−1^ in PBS) was added to each well and the monolayers were incubated for 2 h at 37°C. The medium with MTT was removed and the resulting formazan precipitate was dissolved in DMSO. The extinctions were determined at λ = 540 nm. The percentage of protection was calculated by the formula:


(1) $$\left[\frac{\left(\mathrm{ODexp}.\right)-\left(\mathrm{ODvirus}\;\mathrm{control}\right)}{\left(\mathrm{ODcell}\;\mathrm{control}\right)-\left(\mathrm{ODvirus}\;\mathrm{control}\right)}\right]100$$

where (ODexp.), (ODvirus control), and (ODcell control) indicate the absorbance of the test sample, the virus control and the cell control, respectively. The effective concentration 50 (EC_50_) was determined as the extract concentration inhibiting viral replication by 50%.

### Antibacterial Activity

The following bacterial strains were purchased from the Bulgarian National Bank for Industrial Microorganisms and Cell Cultures (NBIMCC): The Gram-negative bacteria *Acinetobacter calcoaceticus* NBIMCC 3730 and *Klebsiella pneumoniae* NBIMCC 3670, and the Gram-positive bacteria *Bacillus cereus* NBIMCC 1085 and *Staphylococcus aureus* ATCC 25923. The bacterial strains were cultured overnight at 37°C on Muller–Hinton agar (MHA) medium.

#### Disk Diffusion Assay

The extracts were loaded onto sterile paper discs (6 mm in diameter) to obtain final concentration of 5 mg per disc. The 20-ml MHA medium was poured into sterile Petri dishes and inoculated with bacterial suspension (0.1ml/10^8^ CFU ml^−1^). The paper discs with the extracts were placed on the top of agar medium. The Petri dishes were incubated for pre-diffusion at 4°C for 2 h. Sterilized disks loaded with 5% DMSO were used as negative controls. Tetracycline (30 μg/disk) was used as positive reference standard to determine the sensitivity of each bacterial strain tested. The inhibition zones were measured after 24-h incubation period at 37°C. Two replicates and two biological repeats were conducted for each extract. The disk diffusion assay was applied to test the standard phenolic compounds ferulic acid (FA), GA, and Q (Sigma) against the studied bacterial strains.

#### Microdilution Assay

Determination of minimum inhibitory concentration (MIC) was performed by a serial dilution technique using 96-well microtiter plates, according to Wiegand et al. (2008). The experimental inoculum was prepared in two steps as follows: The bacterial suspension was adjusted to McFarland 0.5 and 50 μl were diluted in 10-ml liquid MH medium to a concentration 5 × 10^5^ CFU ml^−1^. The plates were prepared by dispersing 50-μl MH broth into each well. The serial dilutions in the range 40–0.3125 mg ml^−1^ were prepared for each extract directly in the wells. Next, 50 μl of the bacterial inoculum was added into each well reaching a final volume 100 μl. The bacterial growth was detected by measuring the absorbance at λ = 600 nm by ELISA reader (DR-200B, Hiwell Diatek Instruments, Wuxi City, China) at 0 and 24 h after the incubation of plates at 37°C. For positive controls, penicillin G at concentrations in the range rang30–0.235 μg ml^−1^ for Gram-positive bacteria, and bactericidal concertation gentamicin at concentrations in the range 20–0.156 μg ml^−1^ for Gram-negative bacteria were used. The minimum bactericidal concertation (MBC) was determined as the lowest extract concentration inducing 99.9% growth inhibition of the bacterial inoculum after 24 h incubation at 37°C. To additionally confirm the absence of bacterial growth, 10 μl from the defined MBC were plated on agar MH medium.

### Identification and Quantification of Phytochemicals in Methanol Extracts

#### Identification of Phytochemicals Using UHPLC-LTQ OrbiTrap XL

The identification of phytochemicals in methanol Soxhlet extracts (Supplementary Table S2B) was performed by using UHPLC-LTQ OrbiTrap XL according to Banjanac et al. (2017). The UHPLC separation was carried out on an Accela 600 system coupled to the LTQ OrbiTrap XL mass spectrometer (ThermoFisher Scientific, Bremen, Germany). Data were acquired only in the negative ionization mode of the UHPLC-LTQ OrbiTrap MS instrument and iridoid aglycones were not recorded.

#### The UHPLC/qqqMS2 Quantification of Major Phenolics

Phenolics in the methanol extract from dried plant material (Supplementary Table S2B) were chromatographically separated and analyzed at TSQ Quantum Access Max QQQ mass spectrometer (ThermoFisher Scientific, Switzerland), as described by Aničić et al. (2021). The Xcalibur software (version 2.2) was used for instrument control, data acquisition, and analysis. The total amount of compounds in samples was calculated based on the calibration curve of pure compounds and expressed as μg g^−1^ DW. To search the literature on the presence of all these compounds in *Nepeta* or some other species, the SciFinder database^1^ was used.

#### The UHPLC/qqqMS2 Quantification of Major Iridoids

Quantification of iridoids in the methanol extracts from dried plant material was performed in negative ion HESI mode of the UHPLC/qqqMS2 instrument, as described by Aničić et al. (2021). Quantification of 1,5,9-*e*DLA acid was carried out using calibration curves of isolated standards, and the total amount of compounds in the samples was expressed as μg g^−1^ DW. Analytical parameters and validation protocol of the quantitative UHPLC/qqqMS2 method used for the analysis of phenolic and iridoid compounds are provided in Supplementary Table S5.

### Statistical Analysis

All measurements were performed in the period 2019–2021 and included plant material from 3 to 6 biological repeats and technical replicates (*n* ≥ 3). Each biological repeat contained plant material from an average 15 plants (*n* ≥ 15). The results are presented as mean values ± standard errors (SE). To evaluate statistical differences among the treatment variants, one-way ANOVA followed by Holm–Sidak test was performed using Sigma Plot 11.0 software. The differences were considered significant at *p* < 0.05. The data processing for the antiviral assay was done by the Origin 9.1 program. In Sigma Plot, the Pearson's correlation coefficient (*R*) was calculated using average parameter values of the biological repeats for the variants (*n* ≤ 6) with a level of significance 0.05. The principal component analysis (PCA) of the metabolite content in *N. nuda* light variants was performed in R 3.6.3 using *prcomp* function after logarithmic normalization of the data set. PCA plot was done with *ggbiplot* R package.

## Results

### Phylogenetic Position of *N. nuda*

**Figure 1:**
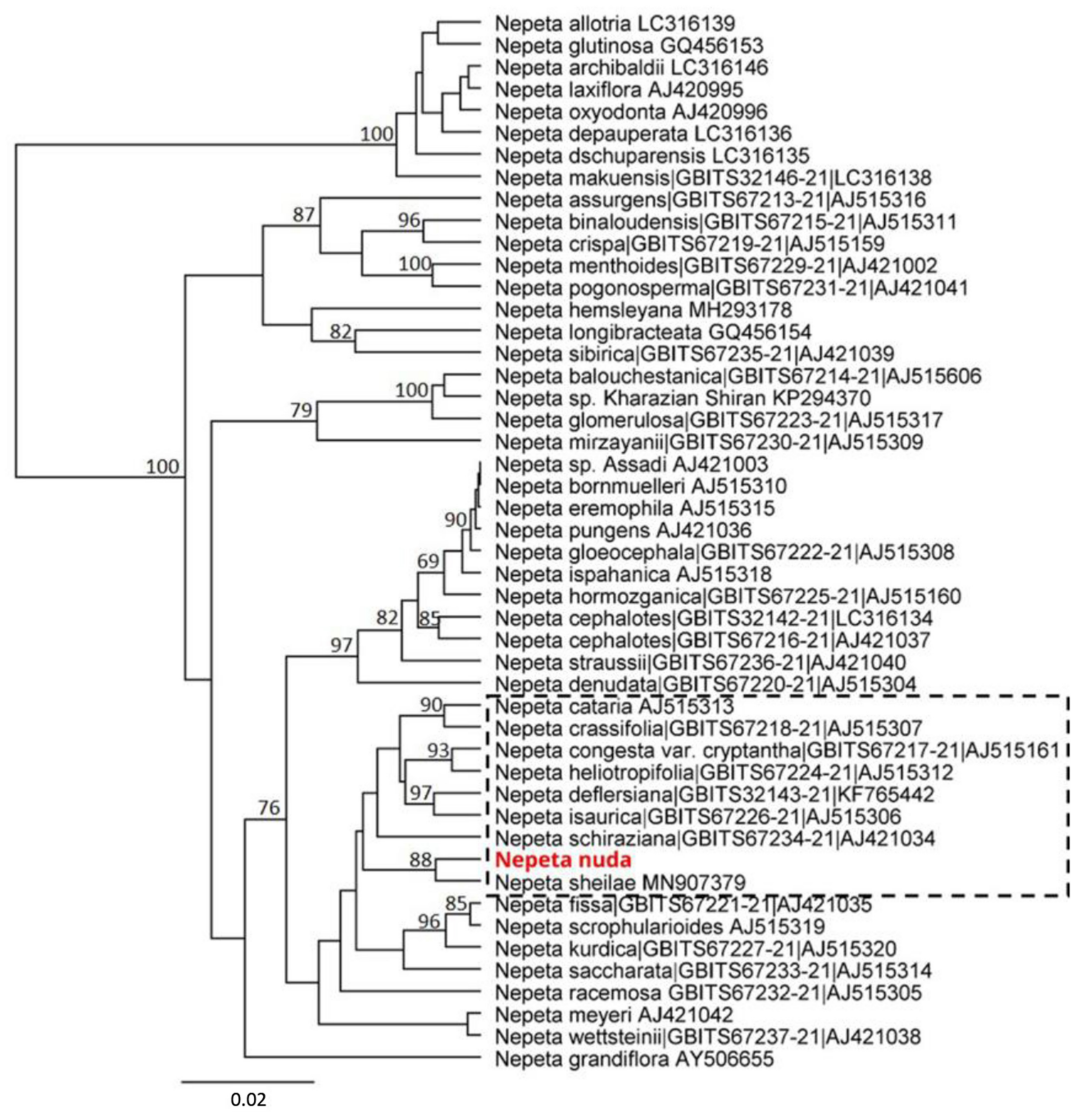
Phylogenetic position of *N. nuda* compared to accessions from BOLD and NCBI GenBank based on nuclear DNA barcoding marker ITS. The tree was constructed using the UPGMA method and Tamura–Nei model. The percentage of replicate trees in which the associated taxa clustered together in the bootstrap test (500 replicates) are shown next to the branches. Bootstrap values below 75% are not shown. The analyzed *N. nuda* sequence is depicted in red bold. Accessions from databases are shown with their respective numbers in BOLD and/or NCBI databases. The branch of accessions close to *N. nuda* is depicted in punctuated rectangle.

**Figure 2:**
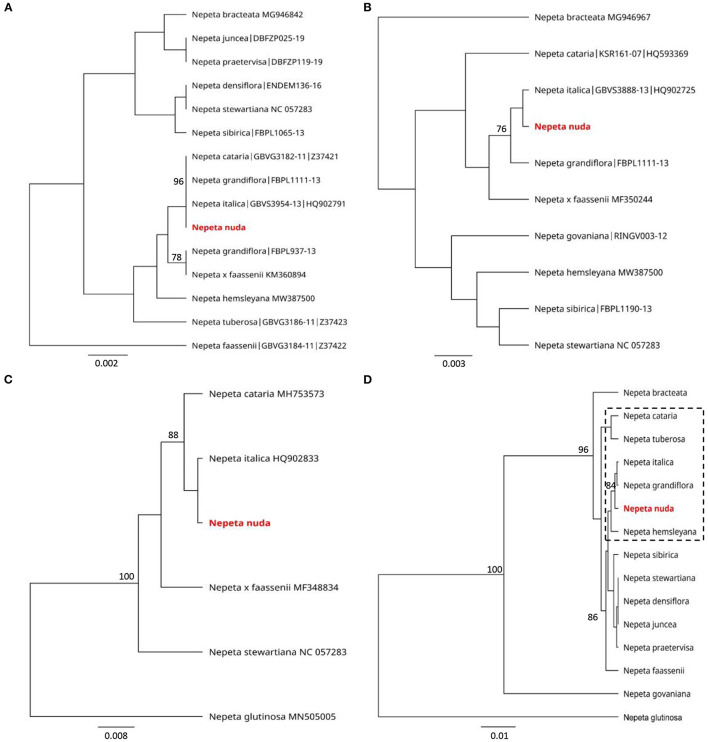
Phylogenetic position of *N. nuda* against accessions from BOLD and NCBI GenBank based on chloroplast DNA barcoding markers *rbcL* **(A)**, *matK* **(B)** and *trnH-psbA* **(C)** and a consensus tree of all three markers **(D)**. The trees were constructed using the UPGMA method and Tamura–Nei model. The percentage of replicate trees in which the associated taxa clustered together in the bootstrap test (500 replicates) are shown next to the branches. Bootstrap values below 75% are not shown. The analyzed *N. nuda* sequence is depicted in red bold. Accessions from databases are shown with their respective numbers in **(A–C)**. In **(D)**, punctuated rectangle points to species in closest proximity to *N. nuda*.

The genetic discrimination of *N. nuda* was a prerequisite step for characterization and validation of its taxonomic position. To achieve this goal, we applied the DNA barcoding method based on the conserved regions ITS and chloroplast (*rbcL, matK, trnH-psbA*) genomes (Supplementary Table S4). The generated DNA barcode sequences were submitted to the BOLD database^2^ (accession number BUL002-22^3^). It should be noted the lack of other accessions in databases belonging to the species *N. nuda*. For the construction of phylogenetic trees, we retreived *Nepeta* accessions from the National Center for Biotechnology Information (NCBI) and BOLD databases, which resulted in a set of 56 sequences for ITS, 44 for *rbcL*, 44 for *matK* and 5 for *trnH-psbA*. Among these sequences, we selected accessions with high homology to *N. nuda* and covered all the available in genebanks species diversity, as the very short fragments were discarded. To make the tree less redundant, one to two accessions per *Nepeta* species from databases were retained, while other accessions with identical sequences and names were discarded. Among the used markers, the ITS-derived sequences for *Nepeta* species were the most enriched in BOLD and NCBI databases, and the phylogenetic tree included 47 *Nepeta* species. The studied *N. nuda* appeared to be most similar to *N. sheilae* (98.7% identity) in a subclade together with *N. schiraziana, N. deflersiana, N. isaurica, N. congesta, N. heliotropifolia, N. crassifolia*, and *N. cataria* (Figure 1). The number of *Nepeta* species for *rbcL*, and *matK* regions was 15 and 10, respectively (Figures 2A,B). For *trnH-psbA*, only NCBI accessions corresponding to six *Nepeta* species were found (Figure 2C). The taxonomic assignment based on chloroplast markers was not congruent with the data for the ITS marker. The data for all three chloroplast DNA barcodes revealed that *N. nuda* falls into one cluster with *N. italica, N. grandiflora, N. hemsleyana, N. cataria*, and *N. tuberosa* (Figure 2D). The DNA barcode *rbcL* showed 100% identity of *N. nuda* to *N. italica, N. grandiflora*, and *N. cataria*. Furthermore, *N. nuda* was very close to *N. italica, N. grandiflora* (99.8%), and *N. cataria* (99.6%) when considering the data from *matK* marker (data are not shown). The *trnH-psbA* marker showed 97.9% and 97.3% similarity to *N. italica* and *N. cataria*, respectively. The results showed the highest identity between *N. nuda* and *N. italica*. The consensus tree of all three chloroplast markers supported the assignment of analyzed *N. nuda* to *N. italica*, and *N. grandiflora* with a bootstrap value of 84% (Figure 2D).

### Environmental Conditions Influence Phenolic Quantity in *N. nuda*

**Figure 3:**
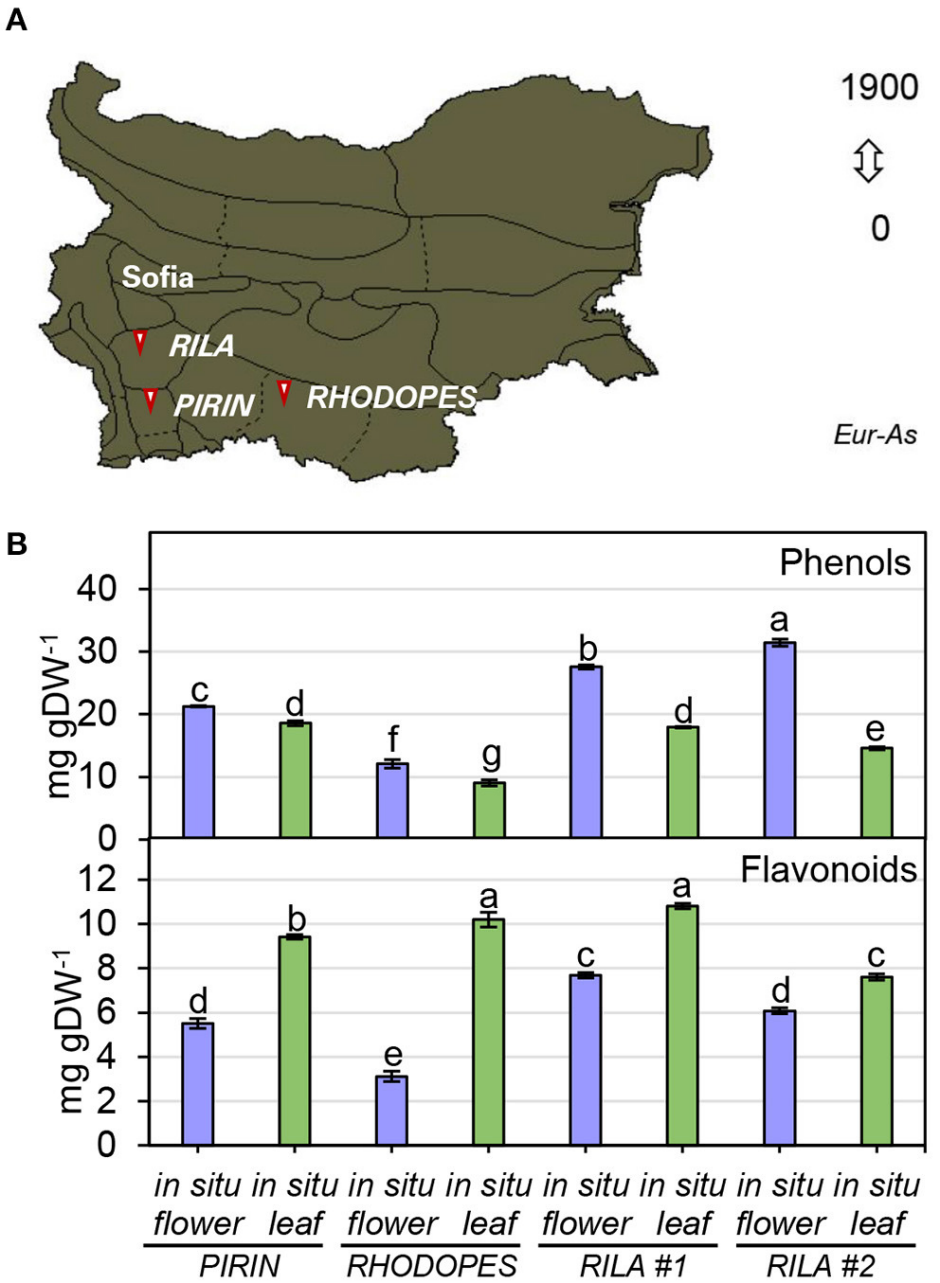
Total phenolics in flowers and leaves of *N. nuda in situ* plants from different habitats. **(A)** Distribution map of *N. nuda* in Bulgaria shown in dark color according to Assyov et al. (2012). Arrows indicate the habitats of the studied *in situ* plants in Pirin, Rhodopes and Rila # 1 and Rila # 2. Samples of vouchers are deposited in the Herbarium of Sofia University “St. Kliment Ohridski,” Sofia, Bulgaria. **(B)** Total content of phenolics in flowers and leaves from *in situ* populations: total phenolic content; total flavonoid content. Mean values ± SE (15 plants; *n* ≥ 3 technical repeats). One–way ANOVA (Holm–Sidak) test was applied to determine the statistical difference between the variants (shown in different letters).

**Figure 4:**
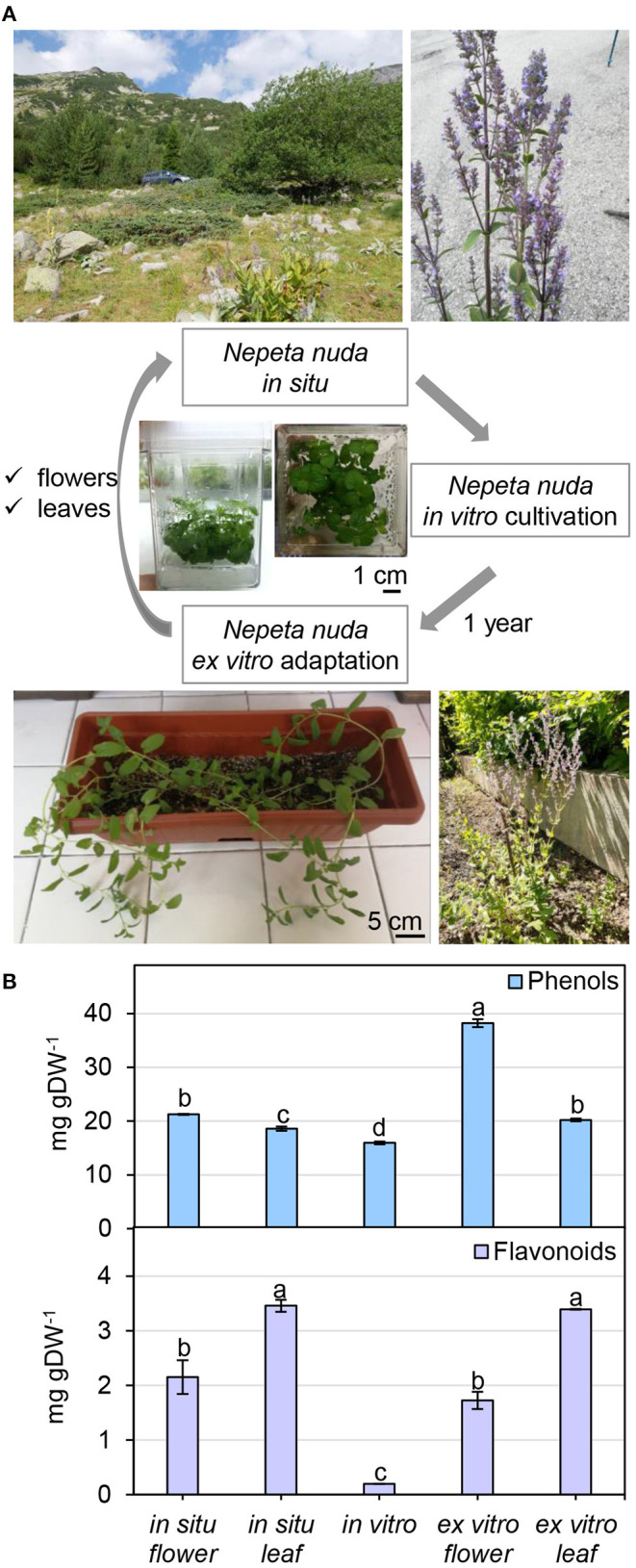
*Nepeta nuda* phenolics in *in situ, in vitro* and *ex vitro* growth conditions. **(A)** *N. nuda* variants: location and photograph of a plant from *in situ* population; *in vitro* plants at 5 weeks; *ex vitro* adapted plants for 1 year. **(B)** Total content of phenolics in flowers and leaves from *in situ* and *ex vitro* plants, and *in vitro* plants: total phenolic content; total flavonoid content. Mean values ± SE (15 plants; *n* ≥ 3 technical repeats). One-way ANOVA (Holm–Sidak) test was applied to determine the statistical difference between the variants (shown in different letters).

To infer the impact of the habitat on the level of phenolic compounds in flowers and leaves, a comparison was performed between *N. nuda* populations collected from three different regions of Bulgaria—Pirin, Rhodopes, and Rila mountains (two samples) (Figure 3A; Supplementary Table S1). The quantity of phenols varied significantly in plants from the distinct habitats. The two samples from Rila were collected at the same place with a difference of growth by 3 weeks which was reflected by a noticeable variation between the respective phenolic content. The plants from Rila were enriched in phenolic compounds compared to the other two populations. As a general trend, more phenolic compounds (up to 30 mg gDW^−1^) were found in flowers compared to leaves (Figure 3B). The opposite trend was observed for the flavonoid content with higher values in the leaves than in the flowers (Figure 3B). The largest quantity of flavonoids (~10 mg gDW^−1^) was detected in the leaves of populations from Rhodopes and Rila. Next, we compared the fluctuation in phenolic content between the flowers and leaves of wild-grown and *in vitro* cultivated plants (Figure 4A). The phenol content was lowest in the *in vitro* control (15.9 mg gDW^−1^) and highest in the flowers of *ex vitro* adapted plants (38.2 mg gDW^−1^) (Figure 4B). Flavonoids were detected in all samples in larger quantities than in the *in vitro* plants (Figure 4B).

### Differential Biological Activities of Wild-Grown and *in vitro* Cultivated Plants

Before testing the biological activities of the extracts, a comparative analysis of the yield using different solvents (water, methanol, ethanol, acetone, and chloroform) was carried out. The highest yield was achieved when water was used for extraction (nearly 37%), and the lowest with acetone (4%) as a solvent (Supplementary Table S2A). The extraction with water and methanol produced the highest phenolic content (nearly 100 mg gExtract^−1^), and the lowest was detected with chloroform (7-mg gExtract^−1^). Flavonoid content was enriched in acetone and ethanol (68- and 24-mg gExtract^−1^, respectively) extracts. The EC_50_ for DPPH-scavenging demonstrated the largest antioxidant activity in water and methanol extracts followed by ethanol, acetone and chloroform (in chloroform the activity was almost lacking). Pearson correlation analysis in Supplementary Table S2A shows a significant correlation between the amount of phenolics and DPPH scavenging activity (*R* = −0.706 with *p* = 0.001). Furthermore, a comparison between the crude methanol extracts of *N. nuda* variants revealed the most effective DPPH radical scavenging (mM Trolox gDW^−1^) in the flowers of wild-grown plants followed by the leaves, whereas a pronounced decline in the *in vitro* cultivated plants was observed (Table 1). In accordance, significant correlation (*R* = 0.943, *p* = 0.0004) was confirmed between the DPPH activity and the phenolic content.

**Table 1:** Antioxidant activity of *N. nuda* crude methanol extracts.

***N. nuda*** **methanol plant DW**	**DPPH radical scavenging(mM Trolox gDW^**−1**^)**
*In situ* flower	128.59 ± 4.81^b^
*In situ* leaf	107.11 ± 2.80^c^
*In vitro*	60.81 ± 1.61^e^
*Ex vitro* flower	206.00 ± 2.80^a^
*Ex vitro* leaf	75.63 ± 0.98^d^

*One-way ANOVA (Holm-Sidak) test was applied to determine the statistical difference between the variants (shown in different letters)*.

Next, the biological activity of *N. nuda* water extracts from the *ex vitro* flowers and leaves and the *in vitro* plants was compared. To evaluate the cytotoxicity of the extracts, the concentrations in the range 0.125–9.0 mg ml^−1^ were applied on MDBK cells (Table 2). The most toxic was the extract from the leaves of *ex vitro* plants (CC_50_ = 5.2 mg ml^−1^) and the least toxic was the water extract from *in vitro* grown plants (CC_50_=7.5 mg ml^−1^). The strong antiviral potential of water extracts from the flowers of *ex vitro* grown *N. nuda* manifested by the inhibition of viral replication and cell survival was dose dependent. The treatment of HHV-1 strain F with the flower water extract was performed simultaneously and 1 h after cell inoculation reaching antiviral cell protection at MNC 81.37% (EC_50_ 0.599 mg ml^−1^) and 65.62% (EC_50_ 1.155 mg ml^−1^), respectively (Table 2). The water extract from the leaves of *ex vitro* grown *N. nuda* also showed substantial antiviral potential (Table 2) but only after simultaneous cell inoculation. At MNC, the cell protection reached 75.18% (EC_50_ 0.725 mg ml^−1^). After the addition of leaf water extracts 1 h after cell inoculation at MNC, the cell protection hardly reached 43.87%. Water extracts from *in vitro* grown plants showed no antiviral activity (Table 2).

**Table 2:** Antiviral activity of *N. nuda* water extracts against HHV-1 (strain F).

**Water extract**	**Cell viability**		**Antiviral activity**					
	**MNC (mg ml^**–1**^)**	**CC_**50**_ (mg ml^**–1**^)**	**The extract added simultaneously with inoculation of cell monolayer**			**The extract added 1 h after inoculation of cell monolayer**		
			**Cell protection (%) when the extracts are added in MNC**	**EC_**50**_ (mg ml^**–1**^)**	**SI**	**Cell protection (%) when the extracts are added in MNC**	**EC_**50**_ (mg ml^**–1**^)**	**SI**
*Ex vitro* flower	1.5	6.4	81.37	0.599	10.68	65.62	1.155	5.54
*Ex vitro* leaf	2	5.2	75.18	0.725	7.17	43.87	n.d.	n.d.
*In vitro*	3	7.5	n.d.	n.d.	n.d.	n.d.	n.d.	n.d.
Positive control								
Acyclovir	0.0312	0.341	97.315	0.00136	250.7	100	0.000609	559.93

*SI (Selective Index)—the ratio of CC_50_ and EC_50_ to determine the extract selectivity to a viral target relative to the cell (SI = CC_50_/EC_50_)*.

*n.d., not detected*.

Further, we tested the antibacterial activity of *N. nuda* extracts against selected Gram (–) and Gram (+) bacteria by three different methods: Disk diffusion, microdilution with the establishment of MIC in liquid bacterial suspension, and lethal MBC by simultaneous plating of extract and bacteria on an agar plate (Table 3; Supplementary Figure S1). The antibacterial activity of *N. nuda* extracts against the tested bacterial strains was confirmed by all the three approaches. Interestingly, the extracts from *in situ* and *in vitro* plants showed similar activity, which suggests that the antibacterial activity of *N. nuda* was not affected by environmental conditions. The disk diffusion assay was suitable for testing of phenolic standards, as its application indicated that the phenolic acids FA and GA had antibacterial activity against the tested bacterial strains, while the flavonoid Q did not have any effect (Supplementary Figure S2).

**Table 3:** Antibacterial activity of *N. nuda* methanol extracts.

***N. nuda*** **methanol extract**	**MIC/MBC (mg ml** ^**−1**^ **)**			
	**Gram (–) bacteria**		**Gram (+) bacteria**	
	***A. calcoaceticus***	***K. pneumoniae***	***B. cereus***	***S. aureus***
*in situ* flower	5/20	2.5/20	0.626/5	5/20
*in situ* leaf	1.25/10	2.5/20	1.25/10	5/20
*in vitro*	5/20	5/20	1.25/10	5/20
Positive controls	MIC/MBC			
penicillin G (μg ml^−1^)	–	–	<0.235	<0.235
gentamicin (μg ml^−1^)	1.25	0.625	–	–

*Values for minimum inhibitory (MIC) and lethal concentration (MBC, minimum bactericidal concentration) are presented as mg of extract per ml (mg ml^−1^)*.

### Environmental Impact on *N. nuda* Metabolites

The profiling of polyphenols and iridoids in *N. nuda* methanol extracts resulted in the identification of 63 compounds that were divided into the following four major groups: (i) Phenolic acids and derivatives (30 compounds); (ii) flavonoid glycosides and aglycones (21 compounds); (iii) iridoid glycosides (11 compounds); and (iv) one compound belonging to another class (Table 4; Supplementary Table S6). In the studied samples, 12 derivatives of hydroxybenzoic acids (1–6, 8, 12, 15, 17, 21, 25) were identified including GA hexosides (1, 3), hydroxybenzoic acid-related compounds (5, 6, 8, 12, 15, 17, 21, 25), protocatechuic acid (2) and vanillic acid (4). The hydroxycinnamic acids were represented by 10 compounds as caffeic acid (20) and its derivatives (7, 9, 10, 13, 27), FA (16) and its derivative (14), RA (18), and its derivative (24). The caffeic acid esters nepetoidin B1 (22) and nepetoidin B2 (28), and the caffeic acid oligomer clinopodic acid A (23) were also found. In addition, two hydroxycoumarins, aesculin (11) and aesculetin (19), were detected. Three polyphenols that are combination of two Danshensu molecules were detected: salvianolic acid C (26), and the derivatives methyl salvianolate C1 (29) and methyl salvianolate C2 (30). The identified flavonoids belonged entirely to the subgroup of flavones with eight luteolin-related compounds (31, 34–37, 41–43), 10 apigenin-related compounds (32, 33, 38–40, 44–47, 49), thymusin (48), cirsimaritin (50), and xanthomicrol (51). Among the identified flavonoid glycosides, except apigenin 7-*O*-hexoside (32), all other derivatives were glucuronides of the flavones apigenin and luteolin. A total of 11 iridoid glycosides were identified as listed in the following: Two nepetanudosides (52, 56), dihydrocornic acid (53), secologanin (54), six epideoxyloganic acid-related compounds (*e*DLA) (55, 57–59, 61, 62), and geniposidic acid (60). The compound 12-*O*-hexosyl-jasmonate (63) was placed in the group “Other”.

**Table 4:** Metabolites identified in *N. nuda*.

**Compound name**	**This study** ***N. nuda*** **subsp**. ***nuda***		**Mišić et al. (2015) *N. nuda***	**Hinkov et al. (2020) *N. nuda* subsp. *nuda***	**Smiljković et al. (2018) *N. nuda***	**Dienaite et al. (2018) *N. nuda***	**Aras et al. (2016b) *N. nuda* subsp. Lydiae**	**Sarikurkcu et al. (2019) *N. nuda* subsp. *glandulifera***
	**UHPLC-LTQ OrbiTrap XL** ^**a**^	**UHPLC/ DAD/qqqMS** ^**b**^	**UHPLC-LTQ OrbiTrap XL**	**NMR**	**LC-DAD-ESI/MSn**	**UPLC-Q/TOF**	**UHPLC-ESI-MS/MS**	**RP-HPLC**
**Phenolic acid derivatives**							
Aesculetin	19							
Aesculin	11	(11)	+					
Benzoic acid methyl ester						+		
Benzoyl tartaric acid	15							
Caffeic acid	20	(20)	+	+		+	+	+
Caffeic acid hexoside 1	9							
Caffeic acid hexoside 2	13							
Caffeic acid hexuronide	7							
Caffeoylmalic acid						+		
Caffeoyl tartaric acid	10							
Chlorogenic acid		(65^*^)	+	+		+	+	+
Cinnamic acid			+	+				
Clinopodic acid A	23							
Coumaric acid							+	
Dicaffeoylquinic acid			+					
Dihydroxybenzoic acid hexoside 1	5							
Dihydroxybenzoic acid hexoside 2	6							
Dihydroxybenzoic acid hexoside 3	12							
Dimethoxy cinnamic acid			+					
Ethyl caffeate	27							
Ferulic acid	16	(16)	+	+		+		+
Feruloyl tartaric acid	14							
Gallic acid		(64^*^)		+				
Gallic acid hexoside 1	1							
Gallic acid hexoside 2	3							
Gentisic acid	8							
Methyl 2-hydroxy-3-(3-hydroxy-4-methoxyphenyl) propanoate	17							
Methyl rosmarinate	24							
Methyl salvianolate C 1	29							
Methyl salvianolate C 2	30							
Nepetoidin B 1	22							
Nepetoidin B 2	28							
*p*-Hydroxybenzoic acid	25							
Protocatechuic acid	2	(2)	+	+	+			+
Salvianolic acid A					+			
Salvianolic acid B					+			
Salvianolic acid C	26							
Umbelliferone						+		
Rosmarinic acid	18	(18)	+	+	+	+	+	n.d.
Syringic acid	21		+			+		
Vanillic acid	4		+	+				
**Other polyphenols**								
Eukovoside					+			
Lithospermic acid					+			
Plantamajoside					+			
Verminoside					+			
**Flavonoids**								
Acacetin			+					
Apigenin	47	(47)	+			+	+	+
Apigenin 7-*O*-(acetyl)hexuronide	40							
Apigenin 7-*O*-(acetyl-caffeoyl) hexuronide	49							
Apigenin 7-*O*-(caffeoyl) hexuronide	39							
Apigenin 7-*O*-(feruloyl)hexuronide 1	45							
Apigenin 7-*O*-(feruloyl) hexuronide 2	46							
Apigenin 7-*O*-(sinapoyl) hexuronide	44							
Apigenin 7-*O*-hexoside	32					+		
Apigenin 7-*O*-hexuronide	33					+		
Apigenin 7-*O*-hexuronide methyl ester	38							
Apigetrin		(66^*^)						
Astragalin		(73^*^)						
Calcelarioside					+			
Chrysoeriol				+				
Cirsimaritin	50	(50)		+				
Eryodictyol		(68^*^)						
Galangin			+					
Hispidulin		(67^*^)						
Isorhamentin		(74^*^)						
Isoquercetin		(72^*^)						
Kaempferol			-				+	
Kaempferol dimethyl ether			+					
Kaempferol monomethyl ether			+					
Kaempferol O-hexoside			+					
Luteolin	42	(42)	+			+	+	
Luteolin-7-O-diglucuronide						+		
Luleolin 7-*O*-(feruloyl) hexuronide 1	41							
Luleolin 7-*O*-(feruloyl) hexuronide 2	43							
Luleolin 7-*O*-hexuronide 1	31							
Luleolin 7-*O*-hexuronide 2	34							
Luteolin 7-*O*-(caffeoyl) hexuronide	37							
Luteolin 7-*O*-(acetyl)hexuronide 1	35							
Luteolin 7-*O*-(acetyl)hexuronide 2	36							
Naringenin		(69^*^)	+					
Quercetin		(70^*^)	+	+				
Quercetin dimethyl ether			+					
Quercetin tetramethyl ether			+					
Quercetin trimethyl ether			+					
Rhamnetin							+	
Rutin		(71^*^)		+				n.d.
Thymusin	48							
Vanillin				+				
Xanthomicrol	51							
**Iridoid glycosides**								
Epideoxyloganic acid 1	59	(59)				+		
Epideoxyloganic acid 2	61	(59)						
Epideoxyloganic acid 3	62	(59)						
Epideoxyloganic acid hexoside 1	55							
Epideoxyloganic acid hexoside 2	58							
Epideoxyloganic acid pentoside	57							
Geniposidic acid	60							
Ligstroside hexoside					+			
Dihydrocornic acid	53							
Nepetanudoside	56							
Nepetanudoside B	52							
Secologanin	54							
**Other compounds**								
12-*O*-hexosyl-jasmonate	63							
Quinic acid		(75^*^)					+	

a *See Supplementary Table S5*.

b *See Figure 4*.

* Based on identified compounds in this work and additional phenolic-related compounds (labeled 64^*^ to 75^*^), in total 21 compounds were selected for comparative quantification between the studied N. nuda samples by the UHPLC/DAD/qqqMS method.

**Figure 5:**
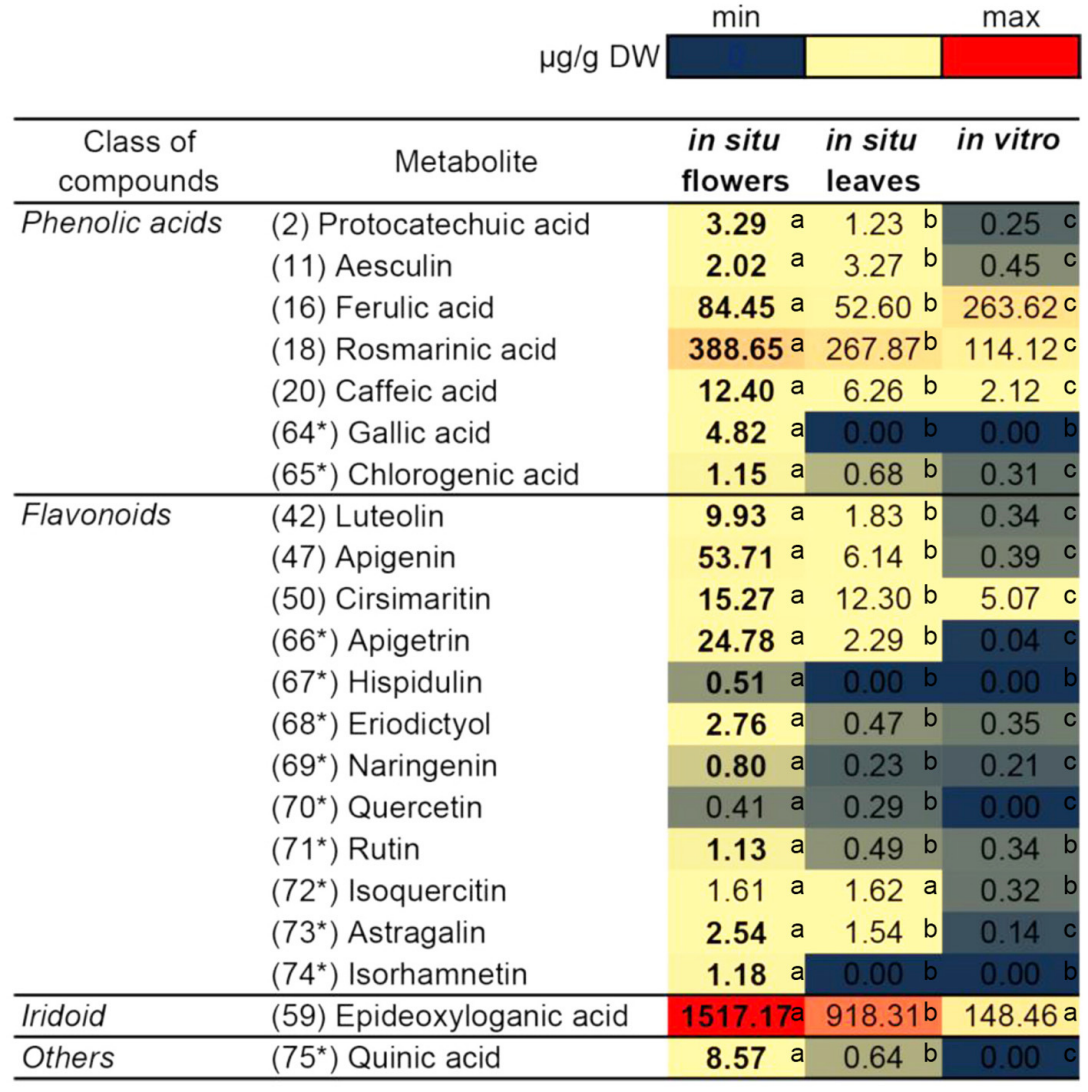
Comparison of the quantity of major compounds among *in situ* flowers, *in situ* leaves and *in vitro* leaves of *N. nuda* by using UHPLC/MS2 analysis. Based on identified compounds in this work and additional phenolic-related compounds (labeled 64^*^ to 75^*^), in total 21 compounds were selected for comparative quantification. Heat map visualization. One-way ANOVA (Holm–Sidak) test was applied to determine the statistical difference between the variants (shown in different letters).

Based on the identified compounds in this work and additional phenolic-related compounds (labeled 64^*^ to 75^*^), 21 compounds in total were selected for comparative quantification between the studied *N. nuda* samples by the UHPLC/DAD/qqqMS method (Figure 5). These 21 compounds were presented by 7 phenolic acids, 12 flavonoids, 1 iridoid, and quinic acid. The following phenolic acid-related compounds were chosen from Supplementary Table S5 and quantified: Protocathechuic acid (2), aesculin (11), FA (16), RA (18), caffeic acid (20), and in addition GA (64^*^) and chlorogenic (5-*O*-caffeoylquinic) acid (65)^*^. The quinic acid (75^*^) was measured as a conjugate with caffeic acid forming the chlorogenic acid. The data highlighted RA (18) and FA (16) as the most abundant compounds followed by caffeic acid (20). Besides identifying flavones shown in Supplementary Table S5, such as luteolin (42); apigenin (47); and cirsimaritin (50), apigetrin (66^*^) and hispidulin (67^*^) were also measured. To confirm whether other flavonoid classes were present in *N. nuda*, more compounds from the class of flavanones were investigated: eriodictyol (68^*^) and naringenin (69^*^); and from the class of flavonols: Q (70^*^), rutin (71^*^), isoquercetin (72^*^), astragalin (73^*^), and isorhamnetin (74^*^). The results indicated that in the used experimental conditions, the flavones were the predominant flavonoids represented mostly by apigenin (47), apigetrin (66^*^), and cirsimaritin (50). The metabolite accumulation under *in situ* and *in vitro* conditions was hardly affected. The significant differences between the flowers and leaves were observed, as the metabolite accumulation in the flowers, particularly of the analyzed flavonoids, was generally enhanced. Most of the detected metabolites had highest levels in the flowers, while in the *in vitro* plants, a general reduction was observed. Strikingly, under *in vitro* conditions, FA (16) was the only compound showing an increased accumulation.

### Light Impact on *N. nuda* Metabolites

**Figure 6:**
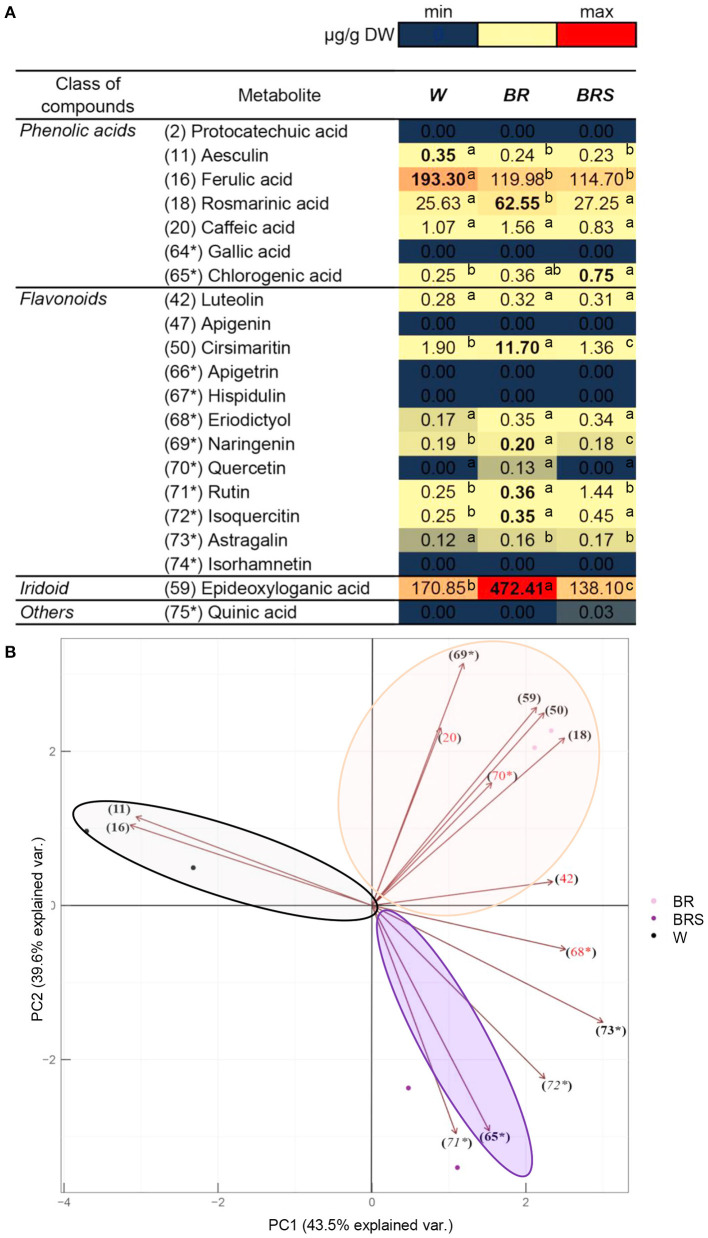
Comparison of the quantity of major compounds between *N. nuda* light variants by using UHPLC/MS2 analysis. Based on identified compounds in this work and additional phenolic-related compounds (labeled 64^*^ to 75^*^), in total 21 compounds were selected for comparative quantification. **(A)** Heat map visualization. One-way ANOVA (Holm–Sidak) test was applied to determine the statistical difference between the variants (shown in different letters). **(B)** PCA analysis. W, white light; BR, blue and red light; BRS, blue and red light with shadow.

To explore the impact of light on metabolite accumulation, we cultivated *in vitro* plants under three different lights: White with normal intensity as a control (W), blue–red with high intensity (BR), and blue–red with low intensity (BRS). The compounds from Figure 5 were quantified and subjected to PCA analysis (Figure 6). The total contribution of both principal components (PC) in the variation of the data set was 83.1%: 43.5% for PC1 and 39.6% for PC2. The formation of PC1 was caused mainly by the differences in light spectrum (W vs.BR). The PC2 formation was closely related to the light intensity (BR vs. BRS). The metabolites were distributed according to the light variant, as the following components were significantly upregulated: The W-specific substances aesculin (11) and FA (16); the BR-specific RA (18), cirsimaritin (50), naringenin (69^*^), and 1,5,9-*e*DLA (59); and the BRS-specific chlorogenic acid (65^*^). There were no significant light-dependent changes in caffeic acid (20), luteolin (42), eriodictyol (68^*^) and Q (70^*^). Rutin (71^*^) and isoquercetin (72^*^) were significantly upregulated by BR in comparison to W, but the effect of BRS was rather variable. Astragalin (73^*^) was upregulated by the blue–red light spectrum range independently from the light intensity. The metabolites protocatechuic acid (2), GA (64^*^), apigenin (47), apigetrin (66^*^), hispidulin (67^*^), isorhamnetin (74^*^), and quinic acid (74^*^) were not detected in the *in vitro N. nuda* samples.

## Discussion

In this study, we used a wide range of methodological approaches to assess environmental effects on the phytoimmune capacity of wild-grown *N. nuda* and *in vitro* plants. Special emphasis was placed on differences in the antioxidant potential, antiviral, and antibacterial activities of this species, and the related secondary metabolites as phenolics and iridoid glycosides. Moreover, the molecular taxonomy of *N. nuda* was evaluated using DNA barcoding approach based on conserved nuclear and chloroplast sequences.

### The DNA Barcoding Assists the Taxonomic Characterization of *N. nuda*

The principle of DNA barcoding is to create a shared source of DNA sequences that can be used to identify taxonomic features (Hollingsworth et al., 2011). The DNA barcoding markers used for the members of Lamiaceae include chloroplast DNA regions, such as *rbcL* (a conserved, coding region), *matK* (a rapidly evolving, coding region) and *trnH-psbA* (a rapidly evolving and length variable intergenic spacer) (Fazekas et al., 2008; Theodoridis et al., 2012). The plastome data has been successfully used to estimate family-wide relationships within Lamiaceae with sufficient resolution at subfamily and tribe levels (Li et al., 2016; Zhao et al., 2021). The previous studies highlight the relevance of nuclear non-coding ITS DNA sequences to infer evolutionary relationships at intrageneric taxonomic ranks of *Nepeta* species often correlating with floral and pollen morphological characters (Jamzad et al., 2003a; Al-Qurainy et al., 2014; Özcan, 2019; Alzeibr et al., 2020). Based on ITS markers, Jamzad et al. (2003a) have shown that *Nepeta* is defined as monophyletic consisting of two large clades with four subclades. In our study, we showed that *N. nuda* belongs to a subclade with the closely related species *N. congesta, N. heliotropifolia, N. deflersiana* and *N. cataria*, and with the closest phylogenetic distance to the recently assigned *N. sheilae* Hedge & R.A. King (Al-Qurainy et al., 2014; Alzeibr et al., 2020). While *N. nuda* belongs to the Orthonepeta section, the morphology of the phylogenetically close *N. sheilae*, endemic to Saudi Arabia, resembles with section Oxynepeta (Alzeibr et al., 2020). The other species from this clade also belong to the section Oxynepeta except for *N. cataria*. They not only have close genetic structure but also share common morphological features, such as corolla shape pattern (Jamzad et al., 2003a). According to Fazekas et al. (2008), three-region DNA barcodes from plastid genome allow sufficient genetic discrimination. The available information from the chloroplast markers allowed us to relate *N. nuda* to *N. italica, N. cataria*, and *N. grandiflora* subclade.

*Nepeta italica* L. is considered phylogenetically and chemotaxonomically close to *N. nuda*, as both species contain 1,8-cineole, which is a major constituent of the essential oil (Baser et al., 2000). Caffeic acid esters, predominantly RA (Janicsák et al., 1999; Pedersen, 2000), nepetoidins A and B (Grayer et al., 2003), and leaf surface flavonoids from the flavone group (cirsimaritin, apigenin, luteolin) (Jamzad et al., 2003b) represent important phenolic chemomarkers for the genus *Nepeta* and the whole subfamily Nepetoidae. In addition, chemotaxonomic phenolic profiling supported the close relation of *N. nuda* to *N. cataria, N. grandiflora* M. Bieb. “Dawn to Dusk,” and *N. mussinii* Spreng. ex Henckel, all belonging to the section Cataria (Mišić et al., 2015). Our results support the data reported by Mišić et al. (2015) and allow us to consider chloroplast markers as more reliable for accurate taxonomic assignment of representatives of the genus *Nepeta*, as compared to ribosomal markers. Furthermore, *N. nuda* displays the highest identity (the mean value of three markers was 99.3%) to *N. italica*. The accessions of the latter that are presented in the phylogenetic trees have Greek origin (Theodoridis et al., 2012). This strongly supports the presence of a distinct genetic pool of *Nepeta* species on the Balkan Peninsula but also implies its evolutionary divergence with the presence of species and/or ecotype variability influenced by eco–geographical and demographic factors. However, further work at larger population scale is required to validate this notion.

### Environmental Factors Affect *N. nuda* Antioxidant Status

The production of iridoids and caffeic acid esters as dominant phenolics in *Nepeta* species was assumed to have evolved simultaneously under natural selection toward providing ecological advantages for efficient adaptation to abiotic and biotic factors (Mišić et al., 2015; Mint Evolutionary Genomics Consortium, 2018). *Nepeta nuda* plants are found mostly in open forest areas and meadows, in the mountains and at subalpine altitudes up to 2100 m (Baden, 1991). Kofidis and Bosabalidis (2008) demonstrated that increasing the altitude led to the development of *N. nuda* glandular trichomes and reduction of the respective essential oils. Besides, the epidermal cells of summer leaves contained phenols, while the autumn leaves did not have these compounds. The flavones (cirsimaritin, luteolin, apigenin, and thymusin) are known to function as UV screens for heat reduction, as antimicrobial agents or insect-feeding deterrents (Jamzad et al., 2003b; Hostetler et al., 2017). In our study, differences in the antioxidant parameters between *N. nuda* plants collected from Pirin (1,850 m), Rhodopes (1,750 m), and Rila (1,150 m) could be linked to different altitudes. Interestingly, the two samples from Rila were collected from the same place, but with a difference of 3 weeks, also showed an obvious change in antioxidant parameters. The phenolic compounds and iridoids play an important role in plant defense, as in *N. nuda* these metabolites act as antioxidants and agents with insect antifeeding, phytotoxic, antiproliferative, antiviral, and antibacterial activities (Table 5). Our data support the presence of these secondary metabolites in the wild-grown plants since phenolics were enriched in natural environment, where the exposure to abiotic and biotic factors considerably affected plant immunity. In general, environmental stimuli induce generation of reactive oxygen species (ROS) that can damage plant cells and their function. As antioxidants, the phenolics neutralize free radicals and prevent lipid peroxidation (Pacifico et al., 2015). Compared to the wild-grown plants, during *in vitro* cultivation plant growth and metabolic activity are reduced (Kapchina-Toteva et al., 2014; Yordanova et al., 2017). In the *in vitro* conditions, the herbicide paraquat is used as an elicitor causing oxidative stress and resulting in subsequent accumulation of *N. nuda* phenolic compounds including RA (Cvetković et al., 2015). ROS can also act in signaling cascades to transmit environmental and developmental signals (Xia et al., 2015). Flavonoids can also modulate the auxin-mediated signaling cascades (Mouradov and Spangenberg, 2014; Hostetler et al., 2017). Upon herbivore attack, wounding-generated ROS induce phytoimmune responses including release of jasmonic acid (JA) and synthesis of salicylic acid (SA) for systemic resistance (Roossinck, 2015). The JA induces production of antifeeding compounds for insects (e.g., phenolic acids and iridoids) and essential oil volatile constituents (monoterpenoids) with repellent effect (Table 5).

**Table 5:** Interrelation between biological activities and metabolites identified in *Nepeta* species.

**Biological activities**	**Phenolics and iridoids in *N. nuda***	**Extract details**	**References**
**Antioxidant activity**			
	Nepetoidin B	Lamiaceae	Grayer et al., 2003
	Nepetalactones	*N. nuda* L. ssp. *Nuda*	Gkinis et al., 2010
	Methyl rosmarinate, clinopodic acid, salvianolic acid A, caffeic acid, syringic acid	*Isodon lophathoides* var. *graciliflorus* (Bentham) H. Hara (Lamiaceae)	Zhou et al., 2014
	Chlorogenic acid, RA, quinic acid	*N. nuda* subsp. *lydiae* methanol, ethanol and water extracts	Aras et al., 2016a,b
	RA, FA, chlorogenic acid, syringic acid, caffeic acid, luteolin derivatives	*N. nuda, N. melissifolia* L. and *N. sibirica* L. water, methanol, acetone extracts	Dienaite et al., 2018
	Chlorgenic acid, FA	*N. nuda* L. subsp. grandulifera Hub.-Mor. and Davis methanol extract	Sarikurkcu et al., 2019
	Phenols and flavonoids	*N. nuda* ethanol extract	Dordević et al., 2019
	Salvianolic acid A and B	water-soluble compounds in *Salvia miltiorrhiza*, Lamiaceae	Zhang et al., 2012; Ma et al., 2019
**Phytotoxic, antifeedant, antiproliferative activities**			
Plant–herbivore and prey–predator interactions	Iridoids		Konno et al., 1999
Allelopathy	Water soluble allelochemicals	*N. nuda* subsp. *nuda* water extract	Dragoeva et al., 2017
Allelopathy	Esculetin		Reviewed in Cheng and Cheng, 2015
Allelopathy, antifeedant	Nepetodin, phenolic acid derivatives	*N. teydea* Webb et Berth. root cultures of induced by *Agrobacterium rhizogenes*	Fraga et al., 2017
Allelopathy	1,8 cineole	*N. nuda* essential oil	Kobaisy et al., 2005
	Pulegone	*N. nuda* subsp. *albiflora* (Boiss.) Gams essential oil	Mancini et al., 2009
	Nepetalactones	*N. nuda* subsp. *albiflora* essential oil	Bozari et al., 2013; Bozok et al., 2017
Antiproliferative	Chlorogenic acid, RA, FA	*N. nuda* water extracts	Dienaite et al., 2018
**Antiviral activity**			
Herpes simplex virus type 1 and type 2 (strain BA)	Na	*N. nuda* ssp. *nuda* methanol, chloroform, water extracts	Todorov et al., 2015; Angelova et al., 2016
Herpes simplex virus type 1, (strain F) and type 2 (strain DD)	Simple phenols and flavonoids	*N. nuda* ssp. *nuda* water extracts	Hinkov et al., 2020; this study
HIV and other viruses	Caffeic acid, lithospermic acid, salvianolic acid A, methyl salvianolate A, protocatehuic aldehyde, apigenin, RA	Lamiaceae	Reviewed in Bekut et al., 2018
**Antibacterial**			
	Iridoids		Konno et al., 1999
*K. pneumoniae, S. aureus*		*N. rtanjensis* Diklić & Milojević essential oil	Stojanović et al., 2005
*K. pneumoniae, S. aureus*	*Trans*-caryophyllene, isopulegone, *cis*-sabinol, and *b*-pinene	*N. nuda* subsp. *albiflora* (Boiss.) Gams essential oil	Alim et al., 2009
*B. cereus, S. aureus*	na	*N. nervosa* Royle & Bentham*, N. rtanjensis* Diklić and Milojević*, N. sibirica* L. (*in vitro* plants) methanolic extracts	Nestorović et al., 2010
No activity against *K. pneumoniae, S. aureus*	na	*N. nuda* subsp. *annua* water, methanol, ethanol extracts	Yildirim et al., 2013
*B. cereus*	na	*N. cataria* L. essential oil	Zomorodian et al., 2012
*S. aureus*	na	*N. praetervisa* methanolic extracts and fractions: chloroform, butanol, hexane	Al-Kahraman et al., 2012
Phytopathogenic bacteria (e.g., *Agrobacterium tumefaciens*)	4aα,7β,7aα-nepetalactone, germacrene, elemol, β-caryophyllene, spathulenol, cubenol	*N. nuda* essential oil	Gormez et al., 2013
*K. pneumoniae, S. aureus*	1,8-cineole	*N. nuda* essential oil	Miladinović et al., 2015
*S. aureus, B. cereus*	Nepetoidin B	*Isodon lophathoides* var. *graciliflorus* (Bentham) H. Hara (Lamiaceae)	Zhou et al., 2014
oral pathogens	RA, verminoside	*N. nuda* tincture	Smiljković et al., 2018
*S. aureus*	Phenols and flavonoids	*N. nuda* ethanol extract	Dordević et al., 2019
B. cereus, S. aureus	Nepetalactones, 1,5,9-ELA, RA	*N. rtanjensis* Diklić and Milojević and *N. argolica* Bory and Chaub. in Bory subsp. *argolica* methanol extracts and pure iridoids	Aničić et al., 2021
*A. calcoaceticus, K. pneumoniae, B. cereus, S. aureus*	FA, gallic acid	*N. nuda* subsp. *nuda* methanol extracts (pretreated with chloroform)	This study

*na, not applicable*.

### Antiviral Activities of *N. nuda* Extracts Against Herpes-Related Viruses

The depressive effects of the *N. sibthorpii* Bentham iridoid epinepetalactone provoke alterations in general behavioral pattern of several animal species, and the role of additional compounds is suggested (Galati et al., 2004). In the *N. nuda* extracts, high content of the iridoid glucoside 1,5,9-*e*DLA (59) in the flowers was detected, which could suggest that this compound is the major iridoid in this species and might be attractive for cats, as well. As an indirect support for this assumption is the inhibition of human alphaherpesvirus type 1 by the *N. nuda* extracts. This antiviral effect could be associated with the feline herpesvirus type 1 (FHV-1) that is a cat alphaherpesvirus causing acute ocular surface disease, dermatitis, respiratory disease, and potentially intraocular disease (Thomasy and Maggs, 2016). However, many antiviral drugs developed for treatment of human herpesviruses have been also used for cat treatment after infection with FHV-1. This suggests that *N. nuda* could produce metabolites with a cat healing power. The metagenomic studies have shown that viruses are abundant in the wild plants, but they are typically asymptomatic, as insects and other herbivores are common vectors in virus transmission (Roossinck, 2015). The existing relationships between plants and viruses have shaped their evolution, and strikingly, the viruses could be mutualists rather than pathogens. For instance, the plant viruses can have positive impacts on plants' ability to cope with biotic and abiotic stress factors. Notably, some viruses use plants as vectors and do not replicate in plants but they are transmitted horizontally through insect feeding on plants. Interestingly, virus-infected plants produce the hormone SA, which counteracts the JA response, and makes plants a better host (Roossinck, 2015). We identified 12-*O*-hexosyl-jasmonate (63) in *N. nuda*, which inhibits JA signaling (Miersch et al., 2008). However, the potential mechanism of *N. nuda* effects and herpes-related viruses requires further investigation. In addition to the possible antiviral properties of iridoids, our data pointed to *N. nuda* phenolic compounds accumulating mainly in flowers of the wild-grown plants.

### Direct and Bacteria-Mediated Phytotoxic Potential of *N. nuda*

Our data on phytochemical potential of *N. nuda* provide additional information about the phytotoxic activity of *N. nuda* extracts (Table 5). The inhibition of plant growth occurs upon competition for common resources, such as nutrients and light (Cheng and Cheng, 2015). Phenolic acids and coumarins act as direct allelopathic agents by inhibiting cell proliferation and the respective growth processes (Table 5). The high antiproliferative activity of *N. nuda* extracts is associated with growth inhibition of different plant species (wheat, cucumber, corn, radish, lettuce, cress, purslane; Table 5). Allelopathic effect could be also indirect and mediated by the inhibition of plant growth-promoting bacteria (PGPB). For instance, *A. calcoaceticus* annotated as PGPB (Kang et al., 2009; Ishizawa et al., 2020), could act through production of extracellular gibberellic acid that stimulates plant elongation as a shade avoidance response (Yang and Li, 2017). It could be assumed that the “sun-plant” *N. nuda* can inhibit such light-competing plant species. Besides, *A. calcoaceticus* can improve cucumber growth by assisting degradation of phenolic allelochemicals, and activating antioxidant enzymes (Wu et al., 2018). Other PGPB, such as *K. pneumoniae* promotes plant growth inducing systemic resistance (Liu et al., 2018), and the soil saprophyte *B. cereus* ensures fungal protection of tomato seedlings (Ramírez et al., 2022). The potentially lethal animal and human pathogen *S. aureus* is directly affected by the phenolic compound SA that reduces the pathogen virulence and attachment to the root surface (Prithiviraj et al., 2005). Our data support the notion that phenolic compounds in *N. nuda* extracts directly inhibited the studied bacterial strains by impeding their growth. Interestingly, in contrast to the antioxidant and antiviral activities, where the *N. nuda* extracts from wild-grown plants showed significantly stronger activities than the *in vitro* plants, the antibacterial activities were nearly identical and did not depend on the environmental conditions. Recent report by Aničić et al. (2021) highlights *Nepeta* iridoids (nepetalactones and 1,5,9-*e*DLA) and the phenolic acid RA in methanol extracts, as potent antimicrobial agents. In our study, the *in vitro* plants had an increased FA content. Besides the literature reports discussing the growth inhibition of *K. pneumoniae* by FA (Lo and Chung, 1999), we tested the pure compound and confirmed its activity against all the four bacterial strains (Supplementary Figure S3). Accordingly, methanol extracts from other *in vitro* cultivated *Nepeta* species also inhibit the growth of *B. cereus* and *S. aureus* (Nestorović et al., 2010).

### Light-Dependent Metabolic Modulation of *N. nuda* During *in vitro* Cultivation

The light composition and intensity are important factors in stimulating plant flowering (Zhiponova et al., 2020a). The observed metabolite enrichment in *N. nuda* flowers led to the use of the previously reported light formula for boosting of flowering (Zhiponova et al., 2020a) in our *in vitro* experiment. Consistently with the natural environmental adaptation of *N. nuda*, the high light intensity stimulated RA (18), cirsimaritin (50), naringenin (69^*^), rutin (71^*^), isoquercetin (72^*^), as well as 1,5,9-*e*DLA (59). Lower light intensity significantly upregulated the chlorogenic acid (65^*^), which supports the existence of a positive correlation between the increased production of this metabolite and the low intensity and combination of blue, red and far–red lights (Chen et al., 2016). Aesculin (11) and FA (16) were specific for the W control variant, which could reflect lack of importance for flowering. Indeed, respective decrease (for aesculin) and slightly higher levels (for FA) of these compounds were detected in the flowers, as compared to the leaves.

### Conclusions and Future Perspectives

This study characterized the impact of environmental factors in modulating the content of bioactive compounds in *N. nuda* that is largely distributed in Bulgaria. DNA barcoding enabled us to genetically discriminate and determine the precise phylogenetic position of *N. nuda* using the available *Nepeta* sequence records in public databases. We provided the first DNA barcode records for *N. nuda* in the BOLD database, thus contributing to the enrichment of global catalogs and specific genetic diversity for the genus *Nepeta* in Bulgaria. These data can be a valuable basis for further species identification and support future studies regarding *Nepeta* genetic variability. Organ specificity of phytoimmunity features of *N. nuda*, such as the antioxidant and antiviral activities, phenolic acids, flavonoids and iridoid glycosides, were analyzed and discussed from an ecophysiological perspective, and their dependence on environmental conditions. Furthermore, this study showed that the light spectrum and intensity are crucial factors affecting the differential accumulation of phenolic acids, flavonoids and iridoids in *N. nuda*. Therefore, these studies could strengthen and facilitate the understanding of *N. nuda* ecology and targeted modulation of its productivity under controlled conditions, thus entailing their potential benefits for agriculture and pharmacology.

The current work provides the platform for further detailed studies on *Nepeta* species in multiple research directions. In point of view of genetic diversity, the study of ecological and taxonomic dynamics within the genus *Nepeta* is of particular interest especially when correlating a genetic ecotype and metabolite activities. It would be of interest to define more aspects of the molecular mechanism regulating phenolics and iridoids levels—effect of environmental signals, gene expression, parallel profiles of other *N. nuda* metabolites. Regarding the biological activities, it would be of interest to identify specific related metabolites with importance for food quality and human health. The application of biotechnological approaches would assist targeted accumulation of compounds by elicitors, as well as enhanced metabolic production by cell cultures and bioreactor.

## Data Availability Statement

The DNA barcoding datasets presented in this study can be found in online repository. The names of the repository and accession numbers can be found in the article and Supplementary Material, respectively.

## Author Contributions

MZ collected the plant material. AT did taxonomic annotation of the plant material and collected botanical information. GB and VV executed DNA barcoding assay. GB and MZ performed the phylogenetic study. ZY and MZ maintained *N. nuda in vitro* cultures. DMa and MR prepared the extracts. DMa and LI did antioxidant analyses. AH, KA, and DT designed and performed antiviral assays. DP and LY designed and tested antibacterial activities. UG and DMi designed and performed UHPLC-LTQ OrbiTrap XL and UHPLC/qqqMS2 assays. GC and MZ performed the light experiments. MP performed the PCA analysis. MZ, DP, AH, VV, AA, DMi, and GB wrote the manuscript. All authors discussed the results and approved the final manuscript.

## Funding

This work was financially supported by the Bulgarian National Science Fund (BNSF), Grant No. KP-06-N56/9/12.11.2021 and by the Ministry of Education and Science of Bulgaria, Project BULCode No. Д01-271/02.10.2020, National Program European Scientific Networks. Infrastructure support was provided by Grant BG05M2OP001-1.002-0012-C01 “Sustainable utilization of bio-resources and waste of medicinal and aromatic plants for innovative bioactive products” co-financed by the European Union through the European Structural and Investment Funds, as well by the Bulgarian Ministry of Education and Science, through Operational Program Science and Education for Smart Growth 2018–2023.

## Conflict of Interest

The authors declare that the research was conducted in the absence of any commercial or financial relationships that could be construed as a potential conflict of interest.

## Publisher's Note

All claims expressed in this article are solely those of the authors and do not necessarily represent those of their affiliated organizations, or those of the publisher, the editors and the reviewers. Any product that may be evaluated in this article, or claim that may be made by its manufacturer, is not guaranteed or endorsed by the publisher.

## Footnotes

^1^ https://scifinder-n.cas.org/?referrer=scifinder.cas.org

^2^ www.boldsystems.org

^3^ https://www.boldsystems.org/index.php/Public_RecordView?processid=BUL002-22

## References

[B1] Aćimović M. Stanković-Jeremić J. Cvetković M. (2020). Phyto-pharmacological aspects of *Nepeta nuda* L.: a systematic review. Nat. Med. Mater. 40, 75–83. 10.5937/leksir2040075A

[B2] Alberti S. (2020). The plant response to heat requires phase separation. Nature 585, 191–192. 10.1038/d41586-020-02442-x 32848237

[B3] Alim A. Goze I. Cetin A. Duran A. (2009). Chemical composition and in vitro antimicrobial and antioxidant activities of the essential oil of *Nepeta nuda* L. subsp. Albiflora (Boiss.) gams. Afr. J. Microbiol. Res. 3, 463–467.

[B4] Al-Kahraman Y. M. S. A. Baloch N. Kakar A. M. Nabi S. (2012). *In-vitro* antimicrobial, insecticidal, antitumor, antioxidant activities and their phytochemical estimation of methanolic extract and its fractions of *Nepeta praetervisa* leaves. Int. J. Phytomedicine 5, 531–536.

[B5] Al-Qurainy F. Khan S. Nadeem M. Tarroum M. Gaafar A. R. (2014). Selection of DNA barcoding loci for *Nepeta deflersiana* Schweinf. ex Hedge from chloroplast and nuclear DNA genomes. Genet. Mol. Res. 13, 1144–1151. 10.4238/2014.February.21.3 24634170

[B6] Alzeibr F. M. A. Ali M. A. Rahman M. O. Al-Hemaid F. Lee J. Kambhar S. V. (2020). ITS gene based molecular genotyping of *Nepeta sheilae* Hedge & R.A. King (Lamiaceae) endemic to Saudi Arabia. Bangladesh J. Plant Taxon. 27, 185–189, 10.3329/bjpt.v27i1.47579

[B7] Angelova P. Hinkov A. Tsvetkov V. Shishkova K. Todorov D. Shishkov S. (2016). Inhibition of human herpes virus type 2 replication by water extract from *Nepeta nuda* L. Acta Microbiol. Bulg. 32, 148–149.

[B8] Aničić N. Gašić U. Lu F. Cirić A. Ivanov M. Jevtić B.. (2021). Antimicrobial and immunomodulating activities of two endemic *Nepeta* species and their major iridoids isolated from natural sources. Pharmaceuticals (Basel) 14:414. 10.3390/ph14050414 33925239 PMC8145025

[B9] Aras A. Bursal E. Dogru M. (2016b). UHPLC-ESI-MS/MS analyses for quantification of phenolic compounds of *Nepeta nuda* subsp. lydiae. J. Appl. Pharm. Sci. 6, 9–13. 10.7324/JAPS.2016.601102

[B10] Aras A. Dogru M. Bursal E. (2016a). Determination of antioxidant potential of *Nepeta nuda* subsp. lydiae. Anal. Chem. Lett. 6, 758–765. 10.1080/22297928.2016.1265467

[B11] Asenov I. (1989). Genus *Nepeta* L. in the *Florae Reipublicae Popularis Bulgaricae Vol. 9*, ed. Velchev V. (Bulgaria: Acad. Sci. Bulgaricae), 374–379.

[B12] Assyov B. Petrova A. Dimitrov D. Vassilev R. (2012). Conspectus of the Bulgarian Vascular Flora. Distribution maps and Floristic Elements, Fourth Revised and Enlarged Edition. Sofia: Bulgarian Biodiversity Foundation.

[B13] Baden C. (1991). Lamiaceae, in Mountain Flora of Greece, Vol. 2, eds. Strid A. Tan K. (Edinburgh: Edinburgh University Press), 66–167.

[B14] Banjanac T. Dragićević M. Šiler B. Gašić U. Bohanec B. Nestorović Živković J.. (2017). Chemodiversity of two closely related tetraploid Centaurium species and their hexaploid hybrid: metabolomic search for high-resolution taxonomic classifiers. Phytochemistry 140, 27–44. 10.1016/j.phytochem.2017.04.005 28448798

[B15] Baranauskiene R. BendŽiuviene V. RagaŽinskiene O. Venskutonis P. R. (2019). Essential oil composition of five *Nepeta* species cultivated in Lithuania and evaluation of their bioactivities, toxicity and antioxidant potential of hydrodistillation residues. Food Chem. Toxicol. 129, 269–280. 10.1016/j.fct.2019.04.039 31029727

[B16] Baser K. Kirimer N. Kurkcuoglu M. Demirci B. (2000). Essential oils of *Nepeta* species growing in Turkey. Chem. Nat. Compd. 36, 356–359.

[B17] Bekut M. Brkić S. Kladar N. Dragović G. Gavarić N. BoŽin B. (2018). Potential of selected Lamiaceae plants in anti(retro)viral therapy. Pharmacol. Res. 133, 301–314. 10.1016/j.phrs.2017.12.016 29258916 PMC7129285

[B18] Blois M. (1958). Antioxidant determinations by the use of a stable free radical. Nature 181, 1199–1200. 10.1038/1811199a0

[B19] Bozari S. Agar G. Aksakal O. Erturk F. A. Yanmis D. (2013). Determination of chemical composition and genotoxic effects of essential oil obtained from *Nepeta nuda* on *Zea mays* seedlings. Toxicol. Ind. Health. 29, 339–348. 10.1177/0748233711433939 22312034

[B20] Bozek M. (2003). Pollen efficiency and foraging by insect pollinators in three catnip (*Nepeta* L.) species. J. Apic. Sci. 47, 19–24.

[B21] Bozok F. Cenet M. Sezer G. Ulukanli Z. (2017). Essential oil and bioherbicidal potential of the aerial parts of *Nepeta nuda* subsp. albiflora (Lamiaceae). J. Essent. Oil-Bear. Plants 20, 148–154. 10.1080/0972060X.2016.1264279

[B22] Brand-Williams W. Cuvelier M. E. Berset C. (1995). Use of a free radical method to evaluate antioxidant activity. LWT 28, 25–30. 10.1016/S0023-6438(95)80008-5

[B23] Chalchat J. Petrovic S. D. Gorunovic M. S. (1998). Quantity and Composition of Essential Oil of the Wild Plant Nepeta nuda L. from Yugoslavia. J. Essent. Oil Res. 10, 423–425. 10.1080/10412905.1998.9700933

[B24] Chang C. C. Yang M. H. Wen H. M. Chern J. C. (2002). Estimation of total flavonoid content in propolis by two complementary colorimetric methods. J. Food Drug Anal. 10, 178–182. 10.38212/2224-6614.2748

[B25] Chen C. C. Agrawal D. C. Lee M. R. Lee R. J. Kuo C. L. Wu C. R.. (2016). Influence of LED light spectra on in vitro somatic embryogenesis and LC-MS analysis of chlorogenic acid and rutin in *Peucedanum japonicum* Thunb.: a medicinal herb. Bot. Stud. 57:9. 10.1186/s40529-016-0124-z 28597418 PMC5430566

[B26] Cheng F. Cheng Z. (2015). Research progress on the use of plant allelopathy in agriculture and the physiological and ecological mechanisms of allelopathy. Front. Plant Sci. 6, 1020. 10.3389/fpls.2015.01020 26635845 PMC4647110

[B27] Cvetković J. Milutinović M. Boljević J. Aničić N. Živković J. N. Živković S.. (2015). Paraquat-mediated oxidative stress in *Nepeta pannonica* L. Bot. Serb. 39, 121–128.

[B28] De Pooter H. L. Nicolai B. De Buyck L. F. Goetghebeur P. Schamp N. M. (1987). The essential oil of *Nepeta nuda*. Identification of a new nepetalactone diastereoisomer. Phytochemistry 26, 2311–2314. 10.1016/S0031-9422(00)84709-3

[B29] Dienaite L. Pukalskiene M. Matias A. A. Pereira C. V. Pukalskas A. Venskutonis P. R. (2018). Valorization of six *Nepeta* species by assessing the antioxidant potential, phytochemical composition and bioactivity of their extracts in cell cultures. J. Funct. Foods 45, 512–522. 10.1016/j.jff.2018.04.004

[B30] Dordević S. M. Stanisavljević D. M. Milenković M. T. Karabegović I. T. Lazić M. L. Nikolova M. T.. (2019). Formulation of refreshing non-alcoholic beverage with extracts of medicinal plants. Prog. Nutr. 21, 620–630. 10.23751/pn.v21i3.7700

[B31] Dragoeva A. Stoyanova Z. Koleva V. Dragolova D. (2017). Allelopathic activity of *Nepeta nuda* L. subsp. nuda water extracts. ASN 4, 46–51. 10.1515/asn-2017-0007

[B32] Dragolova D. Stefanova M. Dimitrova M. Koleva D. Zhiponova M. Kapchina-Toteva V. (2015). *In vitro* cultivation and ex vitro adaptation of *Nepeta nuda* ssp. *nuda*—correlation between regeneration potential, leaf anatomy, and plastid pigments. Bulg. J. Agric. Sci. 21, 1027–1032.

[B33] Dulbecco R. (1952). Production of plaques in monolayer tissue cultures by single particles of an animal virus. Proc. Natl. Acad. Sci. USA 38, 747–752. 10.1073/pnas.38.8.747 16589172 PMC1063645

[B34] Eisner T. (1964). Catnip: Its Raison D'Être. Science 146, 1318–1320. 10.1126/science.146.3649.1318 14207462

[B35] Fazekas A. J. Burgess K. S. Kesanakurti P. R. Graham S. W. Newmaster S. G. Husband B. C.. (2008). Multiple multilocus DNA barcodes from the plastid genome discriminate plant species equally well. PLoS ONE 3:e2802. 10.1371/journal.pone.0002802 18665273 PMC2475660

[B36] Formisano C. Rigano D. Senatore F. (2011). Chemical constituents and biological activities of *Nepeta* species. Chem. Biodivers. 8, 1783–1818. 10.1002/cbdv.201000191 22006710

[B37] Fraga B. M. González-Coloma A. Alegre-Gómez S. López-Rodríguez M. Amador L. J. Díaz C. E. (2017). Bioactive constituents from transformed root cultures of *Nepeta teydea*. Phytochemistry 133, 59–68. 10.1016/j.phytochem.2016.10.008 28340896

[B38] Galati E. M. Miceli N. Galluzzo M. Taviano M. F. Tzakou O. (2004). Neuropharmacological effects of epinepetalactone from nepeta sibthorpii behavioral and anticonvulsant activity. Pharm. Biol. 42, 391–395. 10.1080/13880200490885059

[B39] Gkinis G. Bozin B. Mimica-dukic N. Tzakou O. (2010). Antioxidant Activity of *Nepeta nuda* L. ssp. *nuda* essential oil rich in nepetalactones from Greece. J. Med. Food 13, 1176–11811. 10.1089/jmf.2009.0218 20626246

[B40] Gormez A. Bozari S. Yanmis D. Gulluce M. Agar G. Sahin F. (2013). Antibacterial activity and chemical composition of essential oil obtained from *Nepeta nuda* against phytopathogenic bacteria. J. Essent. Oil Res. 25, 149–153. 10.1080/10412905.2012.751060

[B41] Grayer R. J. Eckert M. R. Veitch N. C. Kite G. C. Marin P. D. Kokubun T.. (2003). The chemotaxonomic significance of two bioactive caffeic acid esters, nepetoidins A and B, in the Lamiaceae. Phytochemistry 64, 519–528. 10.1016/s0031-9422(03)00192-4 12943769

[B42] Handjieva V. Popov S. Evstatieva N. (1996). Constituents of essential oils from *Nepeta cataria* L., *N. grandiflora* M.B. and *N. nuda* L. J. Essent. Oil Res. 8, 639–643. 10.1080/10412905.1996.9701032

[B43] Harley R. M. Atkins S. Budantsev A. L. Cantino P. D. Conn B. J. Grayer R.. (2004). Labiatae, in The Families and Genera of Vascular Plants, Vol. 7, ed. Kadereit J. W. (Berlin: Springer), 167–275.

[B44] Hedge I. C. (1990). Labiatae, in Flora of Pakistan Vol. 192, eds. Ali S. I. Nasir Y. J. (Karachi: University of Karachi), 310.

[B45] Hinkov A. Angelova P. Marchev A. Hodzhev Y. Tsvetkov V. Dragolova D.. (2020). Nepeta nuda ssp. *nuda* L. water extract: Inhibition of replication of some strains of Human Alpha herpes virus (genus Simplex virus) in vitro, mode of action and NMR-based metabolomics. J. Herb. Med. 21:100334. 10.1016/j.hermed.2020.100334

[B46] Hollingsworth P. M. Graham S. W. Little D. P. (2011). Choosing and using a plant DNA barcode. PLoS ONE 6:e19254. 10.1371/journal.pone.0019254 21637336 PMC3102656

[B47] Hostetler G. L. Ralston R. A. Schwartz S. J. (2017). Flavones: food sources, bioavailability, metabolism, and bioactivity. Adv. Nutr. 8, 423–435. 10.3945/an.116.012948 28507008 PMC5421117

[B48] Ishizawa H. Ogata Y. Hachiya Y. Tokura K. I. Kuroda M. Inoue D.. (2020). Enhanced biomass production and nutrient removal capacity of duckweed via two-step cultivation process with a plant growth-promoting bacterium, *Acinetobacter calcoaceticus* P23. Chemosphere 238, 124682. 10.1016/j.chemosphere.2019.124682 31524619

[B49] Jamzad Z. Chase M. W. Ingrouille M. Simmonds M. S. J. Jalili A. (2003a). Phylogenetic relationships in *Nepeta* L. (Lamiaceae) and related genera based on ITS sequence data. Taxon 52, 21–32. 10.2307/3647299

[B50] Jamzad Z. Grayer R. J. Kite G. C. Simmonds M. S. J. Ingrouille M. Jalili A. (2003b). Leaf surface flavonoids in Iranian species of *Nepeta* (Lamiaceae) and some related genera. Biochem. Syst. Ecol. 31, 587–600. 10.1016/S0305-1978(02)00221-1

[B51] Janicsák G. Máthé I. Miklóssy-Vári V. Blunden G. (1999). Comparative studies of the rosmarinic and caffeic acid contents of Lamiaceae species. Biochem. Syst. Ecol. 27, 733–738. 10.1016/S0305-1978(99)00007-1

[B52] Kang S. M. Joo G. J. Hamayun M. Na C. I. Shin D. H. Kim H. Y.. (2009). Gibberellin production and phosphate solubilization by newly isolated strain of *Acinetobacter calcoaceticus* and its effect on plant growth. Biotechnol. Lett. 31, 277–281. 10.1007/s10529-008-9867-2 18931973

[B53] Kapchina-Toteva V. Dimitrova M. Stefanova M. Koleva D. Stefanov D. Kostov K.. (2014). Adaptive changes in photosynthetic performance and secondary metabolites during white dead nettle micropropagation. J. Plant Physiol. 171, 1344–1353. 10.1016/j.jplph.2014.05.010 25046755

[B54] Kilic O. Hayta S. Bagci E. (2011). Chemical composition of essential oil of *Nepeta nuda* L. subsp. *nuda* (Lamiaceae) from Turkey. Asian J. Chem. 23, 2788–2790.

[B55] Kirmizibekmez H. Atay I. Kaiser M. Yesilada E. Tasdemir D. (2011). *In vitro* antiprotozoal activity of extracts of five Turkish lamiaceae species. Nat. Prod. Commun. 6, 1697–1700. 10.1177/1934578x1100601132 22224291

[B56] Kobaisy M. Tellez M. R. Dayan F. E. Mamonov L. K. Mukanova G. S. Sitpaeva G. T.. (2005). Composition and phytotoxic activity of *Nepeta pannonica* L. essential oil. J. Essent. Oil Res. 17, 704–707. 10.1080/10412905.2005.9699037

[B57] Kofidis G. Bosabalidis A. M. (2008). Effects of altitude and season on glandular hairs and leaf structural traits of *Nepeta nuda* L. Bot. Stud. 49, 363–372.

[B58] Kökdil G. Kurucu S. Yildiz A. (1998). Essential oil composition of *Nepeta nuda* L. ssp. *nuda*, Flavour Fragr. J. 13, 233–234. 10.1002/(SICI)1099-1026(1998070)13:4<233::AID-FFJ730>3.0.CO;2-7

[B59] Konno K. Hirayama C. Yasui H. Nakamura M. (1999). Enzymatic activation of oleuropein: a protein crosslinker used as a chemical defense in the privet tree. Proc. Natl. Acad. Sci. USA 96, 9159–9164. 10.1073/pnas.96.16.9159 10430912 PMC17749

[B60] Kozhuharova E. Benbassat N. Getov I. (2014). Ethnobotanical records of not yet documented therapeutic effects of some popular Bulgarian medicinal plants. Emir. J. Food Agric. 26, 647–651. 10.9755/ejfa.v26i7.18200

[B61] Kumar S. Stecher G. Li M. Knyaz C. Tamura K. (2018). MEGA X: molecular evolutionary genetics analysis across computing platforms. Mol. Biol. Evol. 35, 1547–1549. 10.1093/molbev/msy096 29722887 PMC5967553

[B62] Li B. Cantino P. D. Olmstead R. G. Bramley G. L. Xiang C.-L. Ma Z.-H.. (2016). A large-scale chloroplast phylogeny of the Lamiaceae sheds new light on its subfamilial classification. Sci. Rep. 6, 34343. 10.1038/srep34343 27748362 PMC5066227

[B63] Lichman B. R. Godden G. T. Hamilton J. P. Palmer L. Kamileen M. O. Zhao D.. (2020). The evolutionary origins of the cat attractant nepetalactone in catnip. Sci. Adv. 6, eaba0721. 10.1126/sciadv.aba0721 32426505 PMC7220310

[B64] Liu D. Chen L. Zhu X. Wang Y. Xuan Y. Liu X.. (2018). *Klebsiella pneumoniae* SnebYK mediates resistance against heterodera glyines and promotes soybean growth. Front. Microbiol. 9, 1134. 10.3389/fmicb.2018.01134 29910782 PMC5992472

[B65] Lo H. H. Chung J. G. (1999). The effects of plant phenolics, caffeic acid, chlorogenic acid and ferulic acid on arylamine N-acetyltransferase activities in human gastrointestinal microflora. Anticancer Res. 19, 133–139. 10226534

[B66] Ma L. Tang L. Yi Q. (2019). Salvianolic acids: potential source of natural drugs for the treatment of fibrosis disease and cancer. Front. Pharmacol. 10, 97. 10.3389/fphar.2019.00097 30842735 PMC6391314

[B67] Mamadalieva N. Z. Sharopov F. S. Satyal P. Azimova S. S. Wink M. (2016). Chemical composition of the essential oils of some central asian *Nepeta* species (Lamiaceae) by GLC-MS. Nat. Prod. Commun. 11, 1891–1893. 30508359

[B68] Mancini E. Apostolides Arnold N. De Feo V. Formisano C. Rigano D. Piozzi F.. (2009). Phytotoxic effects of essential oils of *Nepeta curviflora* Boiss. *and Nepeta nuda* L. subsp. albiflora growing wild in Lebanon. J. Plant Interact. 4, 253–259. 10.1080/17429140903225507

[B69] Miersch O. Neumerkel J. Dippe M. Stenzel I. Wasternack C. (2008). Hydroxylated jasmonates are commonly occurring metabolites of jasmonic acid and contribute to a partial switch–off in jasmonate signaling. New Phytol. 177, 114–127. 10.1111/j.1469-8137.2007.02252.x 17995915

[B70] Miladinović D. L. Ilić B. S. Kocić B. D. (2015). Chemoinformatics approach to antibacterial studies of essential oils. Nat. Prod. Commun. 10, 1063–1066. 26197552

[B71] Mint Evolutionary Genomics Consortium (2018). Phylogenomic mining of the mints reveals multiple mechanisms contributing to the evolution of chemical diversity in lamiaceae. Mol. Plant 11, 1084–1096. 10.1016/j.molp.2018.06.002 29920355

[B72] Mišić D. Šiler B. Gašić U. Avramov S. Živković S. Nestorović Živković J.. (2015). Simultaneous UHPLC/DAD/(+/–)HESI–MS/MS analysis of phenolic acids and nepetalactones in methanol extracts of nepeta species: a possible application in chemotaxonomic studies. Phytochem. Anal. 26, 72–85. 10.1002/pca.2538 25431035

[B73] Mithöfer A. Boland W. (2012). Plant defense against herbivores: chemical aspects. Annu. Rev. Plant Biol. 63, 431–450. 10.1146/annurev-arplant-042110-103854 22404468

[B74] Mosmann T. (1983). Rapid colorimetric assay for cellular growth and survival: application to proliferation and cytotoxicity assays. J. Immunol. Methods 65, 55–63. 10.1016/0022-1759(83)90303-4 6606682

[B75] Mouradov A. Spangenberg G. (2014). Flavonoids: a metabolic network mediating plants adaptation to their real estate. Front. Plant Sci. 5, 620. 10.3389/fpls.2014.00620 25426130 PMC4226159

[B76] Murashige T. Skoog F. A. (1962). A revised medium for rapid growth and bioassays with tobacco tissue cultures. Physiol. Plant. 15, 473–497. 10.1111/J.1399-3054.1962.TB08052.X

[B77] Nedelkova Y. Dimitrova M. Yordanova Z. Kapchina–Toteva V. (2012). Micropropagation of *Nepeta nuda* ssp. nuda (Lamiaceae)—influence of auxins and cytokinins. Acta Hortic. 955, 269–273. 10.17660/ActaHortic.2012.955.39

[B78] Nestorović J. Misić D. Siler B. Soković M. Glamoclija J. Cirić A. (2010). Nepetalactone content in shoot cultures of three endemic *Nepeta* species and the evaluation of their antimicrobial activity. Fitoterapia 81, 621–626. 10.1016/j.fitote.2010.03.007 20307630

[B79] Özcan T. (2019). Defining phylogenetic relationship of *Nepeta x tmolea* and its parents via DNA barcoding. PhytoKeys 134, 83–96. 10.3897/phytokeys.134.38238 31686955 PMC6821826

[B80] Pacifico S. Galasso S. Piccolella S. Kretschmer N. Panb S.-P. Marciano S.. (2015). Seasonal variation in phenolic composition and antioxidant and anti–inflammatory activities of *Calamintha nepeta* (L.) Savi. Food Res. Int. 69, 121–132. 10.1016/j.foodres.2014.12.019

[B81] Pádure I. (2004). Chorological and ecological aspects of *Nepeta nuda* L. ssp. *nuda* (syn. *N. pannonica* L.) from Lamiaceae—Nepetoideae in Romania. Analele ştiintifice ale Universitátii Al. I. Cuza Iaşi, Tomul L, s. II a. Biologie vegetalá, 65–70.

[B82] Pádure I. M. Mihaiescu D. Badulescu L. Burzo I. (2008). Chemical constituents of the essential oils of *Nepeta nuda* L. subsp. *nuda* (Lamaceae) from Romania. Rom. J. Biol.-Plant Biol. 53, 31–38.

[B83] Pedersen J. A. (2000). Distribution and taxonomic implications of some phenolics in the family Lamiaceae determined by ESR spectroscopy. Biochem. Syst. Ecol. 28:229–253. 10.1016/S0305-1978(99)00058-7

[B84] Prithiviraj B. Bais H. P. Jha A. K. Vivanco J. M. (2005). *Staphylococcus aureus* pathogenicity on *Arabidopsis thaliana* is mediated either by a direct effect of salicylic acid on the pathogen or by SA–dependent, NPR1–independent host responses. Plant J. 42, 417–432. 10.1111/j.1365-313X.2005.02385.x 15842626

[B85] Ramírez V. Martínez J. Bustillos–Cristales M. D. R. Catañeda-Antonio D. Munive J. A. Baez A. (2022). Bacillus cereus MH778713 elicits tomato plant protection against Fusarium oxysporum. J. Appl. Microbiol. 132, 470–482. 10.1111/jam.15179 34137137 PMC9291537

[B86] Ratnasingham S. Hebert P. D. N. (2007). BOLD: The barcode of life data system (http://www.barcodinglife.org). Mol. Ecol. Notes 7, 355–364. 10.1111/j.1471-8286.2007.01678.x 18784790 PMC1890991

[B87] Reed L. J. Muench H. (1938). A simple method of estimating fifty percent endpoints. Am. J. Epidemiol. 27, 493–497. 10.1093/oxfordjournals.aje.a118408

[B88] Roossinck M. J. (2015). Plants, viruses and the environment: Ecology and mutualism. Virology 479–480, 271–277. 10.1016/j.virol.2015.03.041 25858141

[B89] Salehi B. Valussi M. Jugran A. Martorell M. Ramírez-Alarcón K. Stojanović-Radić Z.. (2018). *Nepeta* species: From farm to food applications and phytotherapy. Trends Food Sci. Technol. 80, 104–122. 10.1016/j.tifs.2018.07.030

[B90] Sarikurkcu C. Eskici M. Karanfil A. Tepe B. (2019). Phenolic profile, enzyme inhibitory and antioxidant activities of two endemic *Nepeta* species: *Nepeta nuda* subsp. *glandulifera* and *N. cadmea*. S. Afr. J. Bot. 120, 298–301. 10.1016/J.SAJB.2018.09.008

[B91] Sharma A. Cooper R. Bhardwaj G. Cannoo D. S. (2021). The genus *Nepeta*: traditional uses, phytochemicals and pharmacological properties. J. Ethnopharmacol. 268:113679. 10.1016/j.jep.2020.113679 33307050

[B92] Singleton V. Orthofer R. Lamuela–Raventys R. (1999). Analysis of total phenols and other oxidation substrates and antioxidants by means of Folin–Ciocalteu reagent. Meth. Enzymol. 299, 152–178. 10.1016/S0076-6879(99)99017-1

[B93] Smiljković M. Dias M. I. Stojkovic D. Barros L. Bukvički D. Ferreira I. C. F. R. (2018). Characterization of phenolic compounds in tincture of edible *Nepeta nuda*: development of antimicrobial mouthwash. Food Funct. 9, 5417–5425. 10.1039/c8fo01466c 30280149

[B94] Stojanović G. Radulović N. Lazarević J. Miladinović D. Doković D. (2005). Antimicrobial activity of *Nepeta rtanjensis* essential oil. J. Essent. Oil Res. 17, 587–589. 10.1080/10412905.2005.9699004

[B95] Süntar I. Nabavi S. M. Barreca D. Fischer N. Efferth T. (2017). Pharmacological and chemical features of *Nepeta* L. genus: its importance as a therapeutic agent. Phytother. Res. 32, 185–198. 10.1002/ptr.5946 29044858

[B96] Takeuchi H. Baba M. Shigeta S. (1991). An application of tetrazolium (MTT) calorimetric assay for the screening of anti–herpes simplex virus compounds. J. Virol. Methods 33, 61–71. 10.1016/0166-0934(91)90008-n 1658029

[B97] Tamura K. Nei M. (1993). Estimation of the number of nucleotide substitutions in the control region of mitochondrial DNA in humans and chimpanzees. Mol. Biol. Evol. 10, 512–526. 10.1093/oxfordjournals.molbev.a040023 8336541

[B98] Theodoridis S. Stefanaki A. Tezcan M. Aki C. Kokkini S. Vlachonasios K. E. (2012). DNA barcoding in native plants of the Labiatae (Lamiaceae) family from Chios Island (Greece) and the adjacent Çeşme–Karaburun Peninsula (Turkey). Mol. Ecol. Resour. 12, 620–633. 10.1111/j.1755-0998.2012.03129.x 22394710

[B99] Thomasy S. M. Maggs D. J. (2016). A review of antiviral drugs and other compounds with activity against feline herpesvirus type 1. Vet Ophthalmol. 19, 119–130. 10.1111/vop.12375 27091747 PMC4930706

[B100] Todorov D. Shishkova K. Dragolova D. Hinkov A. Kapchina-Toteva V. Shishkov S. (2015). Antiviral activity of medicinal plant *Nepeta nuda*. Biotechnol. Biotechnol. Equip. 29, S39–S43. 10.1080/13102818.2015.1047215

[B101] Uenoyama R. Miyazaki T. Hurst J. L. Beynon R. J. Adachi M. Murooka T.. (2021). The characteristic response of domestic cats to plant iridoids allows them to gain chemical defense against mosquitoes. Sci. Adv. 7, eabd9135. 10.1126/sciadv.abd9135 33523929 PMC7817105

[B102] Wiegand I. Hilpert K. Hancock R. E. (2008). Agar and broth dilution methods to determine the minimal inhibitory concentration (MIC) of antimicrobial substances. Nat. Protoc. 3, 163–175. 10.1038/nprot.2007.521 18274517

[B103] Wu F. An Y. Q. An Y. Wang X. J. Cheng Z. Y. Zhang Y.. (2018). Acinetobacter calcoaceticus CSY–P13 mitigates stress of ferulic and p–hydroxybenzoic acids in cucumber by affecting antioxidant enzyme activity and soil bacterial community. Front. Microbiol. 9, 1262. 10.3389/fmicb.2018.01262 29963024 PMC6010532

[B104] Xia X. Zhou Y. Shi K. Zhou J. Foyer C. Yu J. (2015). Interplay between reactive oxygen species and hormones in the control of plant development and stress tolerance. J. Exp. Bot. 66, 2839–2856. 10.1093/jxb/erv089 25788732

[B105] Yang C. Li L. (2017). Hormonal Regulation in Shade Avoidance. Front. Plant Sci. 8, 1527. 10.3389/fpls.2017.01527 28928761 PMC5591575

[B106] Yildirim A. B. Karakas F. P. Turker A. U. (2013). *In vitro* antibacterial and antitumor activities of some medicinal plant extracts, growing in Turkey. Asian Pac. J. Trop. Med. 6, 616–624. 10.1016/S1995-7645(13)60106-6 23790332

[B107] Yordanova Z. Rogova M. Zhiponova M. Georgiev M. Kapchina-Toteva V. (2017). Comparative determination of the essential oil composition in Bulgarian endemic plant *Achillea thracica* Velen. during the process of ex situ conservation. Phytochem. Lett. 20, 456–461. 10.1016/j.phytol.2017.03.011

[B108] Zhang H. A. Gao M. Zhang L. Zhao Y. Shi L. L. Chen B. N.. (2012). Salvianolic acid A protects human SH–SY5Y neuroblastoma cells against H_2_O_2_-induced injury by increasing stress tolerance ability. Biochem. Biophys. Res. Commun. 421, 479–483. 10.1016/j.bbrc.2012.04.021 22516750

[B109] Zhao F. Chen Y. P. Salmaki Y.. (2021). An updated tribal classification of Lamiaceae based on plastome phylogenomics. BMC Biol. 19, 2. 10.1186/s12915-020-00931-z 33419433 PMC7796571

[B110] Zhiponova M. Paunov M. Anev S. Petrova N. Krumova S. Raycheva A.. (2020a). JIP-test as a tool for early diagnostics of plant growth and flowering upon selected light recipe. Photosynthetica 58, 399–408. 10.32615/ps.2019.174

[B111] Zhiponova M. Yordanova Z.h. Pavlova D. Rogova M. Dimitrova M. Dragolova D.. (2020b). Importance of phenolics in populations of *Teucrium chamaedrys* (Lamiaceae) from serpentine soils. Aust. J. Bot. 68, 352–362. 10.1071/BT19124

[B112] Zhou W. Xie H. Xu X. Liang Y. Wei X. (2014). Phenolic constituents from *Isodon lophanthoides* var. graciliflorus and their antioxidant and antibacterial activities. J. Funct. Foods 6, 492–498. 10.1016/j.jff.2013.11.015

[B113] Zomorodian K. Saharkhiz M. J. Shariati S. Pakshir K. Rahimi M. J. Khashei R. (2012). Chemical composition and antimicrobial activities of essential oils from *Nepeta cataria* L. against common causes of food–borne infections. ISRN Pharm. 2012:591953. 10.5402/2012/591953 22779012 PMC3385634

