# Supplementary material for: Catmint (Nepeta nuda L.) Phylogenetics and Metabolic Responses in Variable Growth Conditions
Source: Front Plant Sci. 2022 May 16;13:866777. doi: 10.3389/fpls.2022.866777 (PMC9150856; doi:10.3389/fpls.2022.866777)
Supplement: Supplementary file 1 [file Data_Sheet_1.pdf]

Supplementary Material

The Supplementary Material includes:

1. Supplementary Methods. Extraction Preparation
2. Figures S1, S2
3. Tables S1, S2, S3, S4, S5, S6
4. References

Supplementary Methods

Extracts Preparation

For comparison of solvents efficiency, dried leaves from *N. nuda* plants grown *in situ* were ground into a powder and subjected to various extractions using different solvents (in ratio 1 g DW in 10 ml solvent), such as water, methanol, ethanol, acetone and chloroform at 60°C±5°C (**Table S2A**). Extraction procedure by maceration was applied for water and ethanol in thermostat at 60°C for 24 h, and the respective incubation with acetone was performed at room temperature due to the fast evaporation. Further, the water extracts were filtered through filter paper, and the alcoholic extracts – through glass filter with vacuum. The methanol Soxhlet extracts were prepared with the Soxhlet apparatus where 3 g dried plant material was placed in a cellulose cartridge (Whatman ©) and subjected to extraction with 30 ml of chloroform until complete decolorization at 62°C. After removal of the non-polar compounds, 30 ml of methanol were used to extract the polar substances at 65°C until complete discoloration. As a next step, the solvents were evaporated: for aqueous extraction, frozen filtrate was lyophilized by using freeze-dryer (Alpha 1-2 LDplus, Martin Christ Gefriertrocknungsanlagen GmbH, Osterode am Harz, Germany) at -65°C; for alcoholic extractions (ethanol and methanol), filtrates were evaporated under vacuum using rotary evaporator (VWR, IKA, RV 10 D S93, Staufen, Germany) at 40°C. The dried extracts were stored at -20°C. The yield of each extract variant was estimated as the weight of extract per g of plant DW in percentages. For analyses of phenolics and antioxidant activity, the extracts were dissolved in methanol.

Crude methanol extracts from dried plant material (10 mg ml^{-1}) were prepared by homogenization and ultrasound (**Table S2B**). After centrifugation for 10 min at 13000 rpm, the supernatant was taken for further analyses.

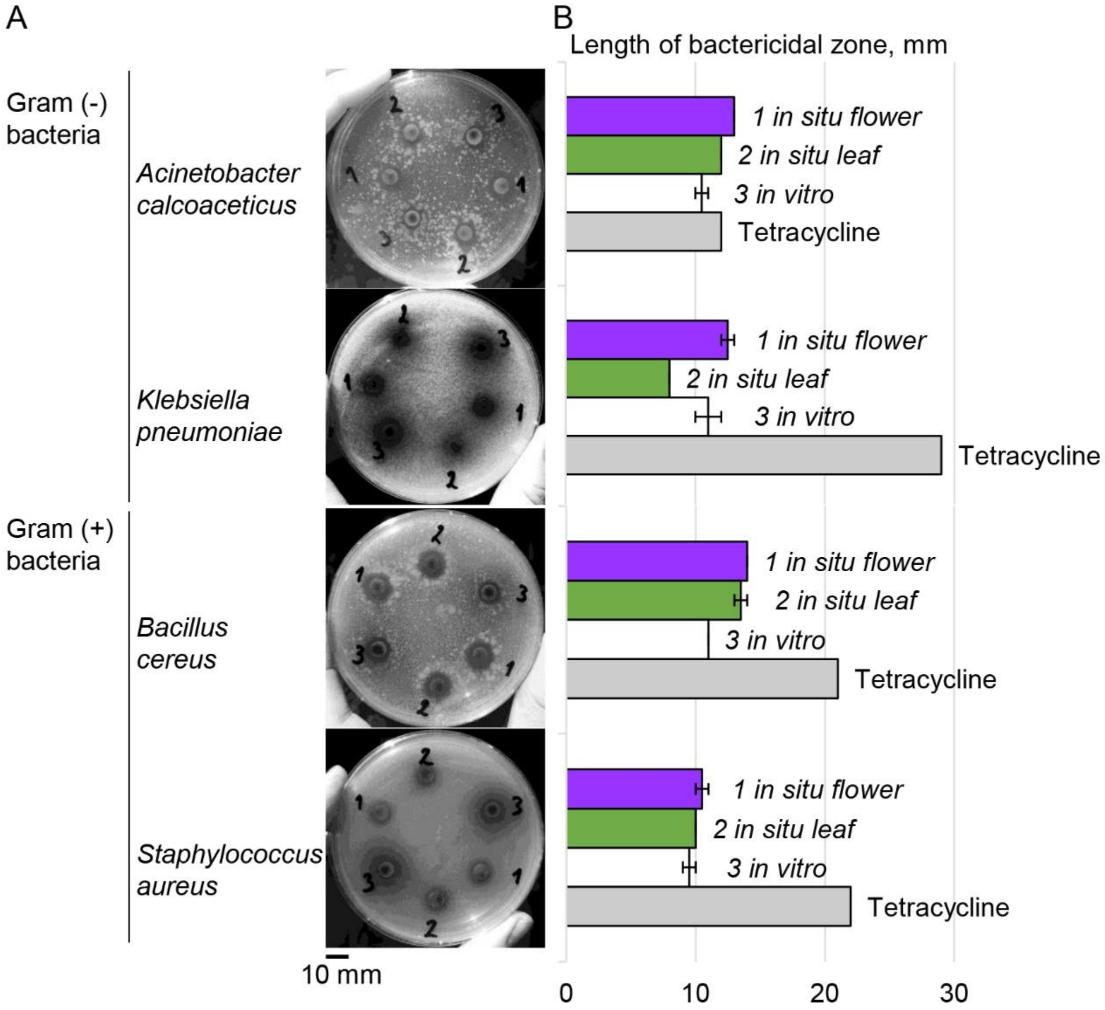

Figure S1. Antimicrobial activity of *N. nuda* extracts (1-*in situ* flower; 2-*in situ* leaf; 3-*in vitro*) by the disk diffusion assay. **(A)** Photos of the petri dishes. **(B)** Quantification of the respective length of the bactericidal zone observed around the discs with extracts resolved in 5% DMSO (the solvent did not affect bacterial growth). Tetracycline positive control is also indicated.

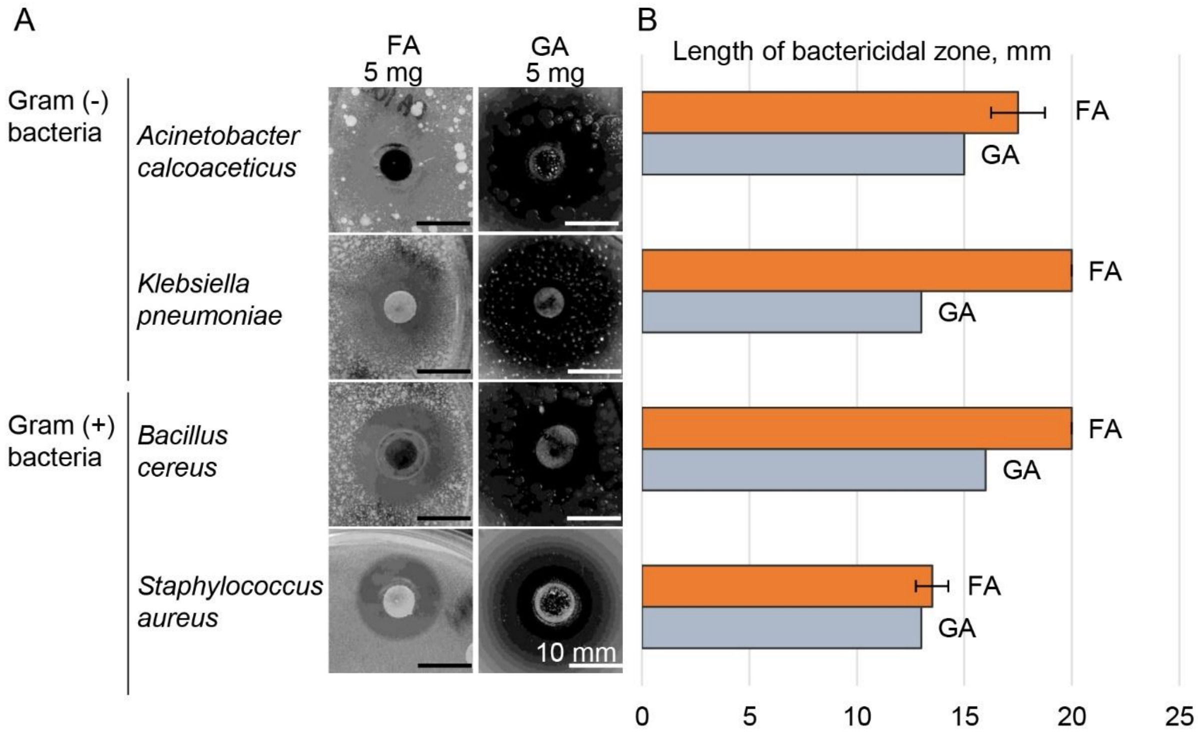

Figure S2. Antimicrobial activity of phenolic compounds by the disk diffusion assay. **(A)** Photos of the petri dishes are zoomed. **(B)** Quantification of the respective length of the bactericidal zone observed around the discs with phenolic compounds resolved in 100% DMSO is shown (solvent did not affect bacterial growth). The tested quercetin did not show any effect. FA – ferulic acid, GA – gallic acid.

Table S1. Locations and respective samples of vouchers deposited in the Herbarium of Sofia University "St. Kliment Ohridski” (SO), Sofia, Bulgaria

Variant *N. nuda*	Region in Bulgaria [coordinates]	Collection Time	Altitude (m)	Voucher (SO)	Reference
*in situ Pirin**	Pirin (between Banderishka meadow and Vihren hut) [41.78166131664765, 23.437755639865717]	3.08.2019 flowering stage	~1850	108017	-
in situ Rhodopes	Rhodopes (near Persenk hut) [41.84679506025972, 24.550550538636827]	9.08.2019 flowering stage	~1750	108016	-
in situ Rila #1	Rila #1 (nearby Rila Monastery) [42.13411930337945, 23.340106069318413]	07.2019 flowering stage	~1150	108014	-
in situ Rila #2	Rila #2 (nearby Rila Monastery) [42.13411930337945, 23.340106069318413]	07.2019 flowering stage	~1150	108015	-
*in vitro**	Lozen mountain, Sofia [42.58472172710655, 23.51675240261395]	5-week-old plantlets	-	105807	Dragolova et al., 2015
*ex vitro**	Pancharevo, Sofia [42.6049869302311, 23.394794039514327]	28.06.2020 flowering stage	~750		

* Material used for comparison between wild-grown and *in vitro N. nuda*.

Table S2. Information about used extracts

(A) Efficacy of tested extract solvents.

N. nuda Extract	Extraction	Extract Yield (%)	Phenols (mg GAE $gExtract^{-1}$)	Flavonoids (mg GAE $gExtract^{-1}$)	EC_{50} DPPH Radical Scavenging (μg ml^{-1}Extract)
Water	Thermostat at 60°C, 24 h	36.95 ± 2.74^{a}	104.69 ± 1.44^{a}	35.53 ± 0.10^{c}	23.53 ± 0.92^{a}
Methanol*	Soxhlet (after Chloroform) at 70°C, until discoloration	10.57 ± 0.95^{c}	100.43 ± 1.80^{a}	23.73 ± 0.61^{e}	18.59 ± 1.34^{a}
Ethanol	Thermostat at 60°C, 24 h	8.80 ± 0.90^{c}	43.76 ± 1.99^{b}	53.07 ± 0.44^{b}	70.81 ± 3.02^{b}
Acetone	Incubation at RT, 24 h	4.00 ± 0.23^{d}	24.50 ± 0.98^{c}	67.76 ± 0.70^{a}	221.28 ± 35.44^{c}
Chloroform*	Soxhlet at 60°C, until discoloration	13.93 ± 1.94^{b}	7.19 ± 0.09^{d}	33.77 ± 0.23^{d}	1284.04 ± 389.71^{d}

*The same plant material was extracted first with chloroform, and after with methanol. For analyses, all extracts were dissolved in methanol. One-way ANOVA (Holm-Sidak) test was applied to determine the statistical difference between the variants (shown in different letters).

(B) *N. nuda* extracts preparation for biological activities and phytochemical analyses

Purpose	*N. nuda* Extracts Preparation			
Method Section	Solvent	Extraction	DW	Reference
2.3&2.4 Total phenolics quantity and DPPH activity	methanol	1) Homogenization with the solvent followed by ultrasound, and analysis of the supernatant.	crude extract	-
2.5 Antiviral activity	water	1) Thermostat at 40°C for 24 h;<br>2) The dry extract was resolved in water with 5% DMSO to a known concentration and sterilized by filtration (Millipore, 0.45 μm).	extract	Hinkov et al., 2020
2.6 Antibacterial activity	methanol	1) Soxhlet (after Chloroform) at 70°C, until discoloration;<br>2) The dry extract was dissolved in water with 5% DMSO to a known concentration and sterilized by filtration (Millipore, 0.45 μm).	extract	Valyova et al., 2011
2.7.1 UHPLC-LTQ OrbiTrap XL	methanol	1) Soxhlet (after Chloroform) at 70°C, until discoloration;<br>2) The dry extract was dissolved in methanol.	extract	Aničić et al., 2021
2.7.2 UHPLC/DAD/qqqMS	methanol	1) Dry plant material was extracted with methanol.	crude extract	Aničić et al., 2021

Table S3. Oligonucleotide primers used for amplification of DNA barcode regions

Barcode Region	Primers	Primer Sequences 5′-3′	PCR Conditions	
**ITS**	ITS5a-F	CCTTATCATTTAGAGGAAGGAG	94℃ 5 min<br>94℃ 30 s<br>50℃ 30 s<br>72℃ 1 min, 35x<br>72℃ 5 min	Stanford et al., 2000
	ITS 4	TCCTCCGCTTATTGATATGC		White et al., 1990
rbcL	rbcLa-F	ATGTCACCACAAACAGAGACTAAAGC	94℃ 4 min<br>94℃ 30 s<br>55℃ 30 s<br>72℃ 1 min, 35x<br>72℃ 10 min	Levin et al., 2003
	rbcLajf634R	GAAACGGTCTCTCCAACGCAT		Fazekas et al., 2008
matK	matK_390F	CGATCTATTCATTCAATATTTC	94℃ 4 min<br>94℃ 30 s<br>51℃ 60 s<br>72℃ 60 s, 40x<br>72℃ 4 min	Cuenoud et al., 2002
	matK_1326r	TCTAGCACACGAAAGTCGAAGT		
trnH-psbA	psbA-trnH	CGCGCATGGTGGATTCACAATCC	94℃ 4 min<br>94℃ 30 s<br>55℃ 30 s<br>72℃ 1 min, 35x<br>72℃ 7 min	Tate and Simpson, 2003
	psbA-3F	GTTATGCATGAACGTAATGCTC		Sang et al., 1997

Table S4. Sequences information for DNA barcoding of *N. nuda*

**Barcode Region**	**Sequence 5'-3' of *Nepeta nuda*, sequence length (bp)**	**BOLD ID**
ITS<br>*593 bp*	CTGCGGAAGGATCATTGTCGAGACCTGCAAAGCAGACCGCGAACACGTAACCAACGCAATTCCCGGCCGGCGCGC<br>TCCCCCGGGACGCGCCGCCCGGGCTAACTAACCCCGGCGCGGAATGCGCCAAGGAAAACCGAACGAAGCGACCGA<br>CCCCCGCGCCCCGTCCGCGGAGCGCGCGGGGGACCGGCCGTCTATCTGAATGTCATAACGACTCTCGGCAACGGA<br>TATCTCGGCTCTCGCATCGATGAAGAACGTAGCGAAATGCGATACTTGGTGTGAATTGCAGAATCCCGTGAACCA<br>TCGAGTCTTTGAACGCAAGTTGCGCCCGAAGCCGTCAGGCCGAGGGCACGTCTGCCTGGGCGTCACGCATCGCGT<br>CGCCCCCCTCCCCTCGCACTGCGCGGGAGAGGCGGGGCGGACATTGGCCCCCCGTGCGCCCCGGCGCGCGGCCGG<br>CCCAAATGTGATCCCCCGGCGACTCGTGTCGCGACGAGTGGTGGTTGAAATCCTCAATCTCGCAGCGTCGCGCCC<br>CCGTGTCGTCCGGACGGGCATCAACGAACGACCCAACGGTGCCTTGCACCATCGACCGCGACCCCAGG	BUL001-22.ITS
rbcL<br>*630 bp*	ACAGAGACTAAAGCAAGTGTTGGATTCAAAGCGGGTGTTAAAGAGTACAAATTGACTTATTATACTCCTGAATAC<br>GAAACCAAAGATACTGATATCTTGGCAGCATTCCGAGTAACTCCTCAGCCTGGAGTTCCGCCTGAAGAAGCAGGG<br>GCCGCGGTAGCTGCCGAATCTTCTACCGGTACATGGACAACTGTGTGGACCGATGGACTTACCAGCCTTGATCGT<br>TACAAAGGGCGATGCTACCACATTGAGCCCGTTCCTGGAGAAAAAGATCAATATATCTGTTATGTAGCTTACCCT<br>TTAGACCTTTTTGAAGAAGGTTCTGTTACTAACATGTTTACTTCCATTGTAGGAAATGTATTTGGATTCAAAGCC<br>CTACGTGCTCTACGTCTGGAAGATCTGCGAGTTCCTCCTGCTTATATTAAAACTTTCCAAGGCCGCCTCATGGG<br>ATCCAAGTTGAGAGAGATAAATTGAACAAGTACGGTCGTCCTCTGCTGGGATGTACTATTAAACCCAAATTGGGG<br>TTATCTGCTAAAAACTATGGTAGAGCGGTTTATGAATGTCTTCGCGGTGGACTTGATTTTACCAAAGATGATGAG<br>AACGTGAACTCCCAGCCATTTATGCGTTGG	BUL001-22.rbcL
matK<br>*819 bp*	TGTTAGAGATACTAATACCTCGCTCTGTTCATGTGGAAATCTTGATTCAAACTCTTCGCCATTGGGTAAAAGATG<br>TTTCTTCTTTCCATTTATTAGGGGTATTTCTCAAGGAATATTGGAATTGGAATAGTCTTCTTACTCCAAAGAAAG<br>TCAGCTTTTCTTTGTCAAAAAGAAATCAAAGGTTATTTTTTTTCTTATATAATTCTCATGTATGTGAATACGAAT<br>CTATTTTCGTCTTTCTACGTAACCAATCTTTTCATTTACGATCAACATCTTCTGGACTTCTTCTTGAAAGAATCT<br>ATTTCTCTATAAAAAGAGAACGTCTTGTGAACGTCTTTGTTAAGGATTTTAGGGCGAACCTGTGGTTAGTCGAGG<br>AACCCTGCATGCATTCTATTAGGTATCAAAGAAAATGCATTCTGGCTTCCAAGGGGACATCCCTTTTCATGAATA<br>AATGGAAATTTTATCTTGTCACTTTTTGGCAATGGCATTTTTCGGTGTGGTTTCATCCAAGAAGGATTTGGATAA<br>ACCAACTTTCCAAGCATTCCCTTGCAATTTTGGGCTATCTTTCAAACGTGCAAATCAACCCTTCCGTGGTACGGA<br>GTCAAATTCTAGAAAATTCATTTTTAATCAATAATGCTATTAAGAAGCTCGATACCCTTGTTCCAATTATTCCTC<br>TGATTTCAGAATTTGCTAAAGCTAAATTTTGTAACGTATTGGGGAATCCCATTAGTAAGCCGATTCGGGCTGAGT<br>TATCAGATTCTAATATTATTGATCGATTTGTGCGTATATGCAGAAATATTTCTCATTATCATAGCGGAT	BUL001-22.matK
trnH-psbA<br>*450 bp*	GGGATTCACAATCCACTGCCTTGATCCACTTGGCTACATCCGCCCCTCTACTTCTACTATTACTATAATTATAAT<br>AAAAATTAGAATAAATAAAATTCTATTAAATATTATTTTATTTTTTATTTAGGAATTCCATTTTTTTAGAAATTT<br>TAAAAGAAAAGATGGAATTTCCATCTTTTTCTCTCTTCTTTAATTCTGTAATAAAAAGAAAAATTCTAGGTAAGT<br>CCAATACTACTAGAAAATATAGAAAACGACTCCTAATTGAAAAGAAAATAAAGGAGCAAGAAACCCTTTCTTGCT<br>TGTTCTATCAAAAGAGGGTTTCTTGCTCCTTATATTTATCTATTCTATATTTTATTTTCAAATAACTCCTACACA<br>CTAAGAACAAGTCTTAGCCGTTTGTTGGAGCTTCGATAGCAGCTAGGTCTAGAGGGAAGTTATGAGCATTACGTT	BUL001-22. trnH<br>-psbA

Table S5. The analytical parameters and validation protocol for quantitative UHPLC/qqqMS2 method.

Compounds	t_R, min	Parent Ion, *m/z*	Product Ions, *m/z* (Collision Energy, eV)	Linear equations, (Y = A + BX)	Linearity, R^2	LOD, mg/L	LOQ, mg/L
Protocatechuic acid	4.23	153.071	109.20 (16); 91.17 (30)	Y = -18147.4+1877190*X	0.9991	0.04	0.12
Aesculin	4.80	339.100	177.10 (25); 133.10 (44)	Y = -90808.3+4791970*X	0.9954	0.08	0.28
Ferulic acid	6.87	193.035	178.04 (15); 134.06 (19)	Y = -16429.5+2265025*X	0.9975	0.07	0.24
Chlorogenic acid	5.18	353.103	191.28 (25)	Y = 84.1362+2519120*X	0.9960	0.06	0.21
Rosmarinic acid	7.17	359.061	197.02 (19); 161.00 (21)	Y = 83696.5+3960747*X	0.9912	0.11	0.35
Gallic acid	2.26	169.100	125.14 (15); 79.36 (23)	Y = -23396.2+5186260*X	0.9905	0.12	0.41
Caffeic acid	5.88	179.004	135.06 (18); 107.13 (18)	Y = -117581+5582020*X	0.9917	0.11	0.38
Luteolin	8.07	285.035	151.03 (28); 133.06 (36)	Y = -116789+5344055*X	0.9980	0.06	0.18
Apigenin	8.93	269.032	225.09 (23); 117.07 (43)	Y = -116048+4509550*X	0.9978	0.06	0.20
Cirsimaritin	10.06	313.010	298.05 (29); 283.04 (41)	Y = -327007+1478640*X	0.9972	0.07	0.22
Apigetrin	6.91	431.004	268.03 (36); 239.11 (53)	Y = 17587.3+3619030*X	0.9984	0.05	0.17
Hispidulin	9.08	299.012	284.09 (25); 256.06 (39)	Y = -510029+2841240*X	0.9982	0.05	0.18
Eriodictyol	8.06	286.974	150.93 (19); 125.06 (21)	Y = -87389.8+3838327*X	0.9978	0.06	0.19
Naringenin	8.84	271.077	151.07 (19); 119.10 (25)	Y = -53519.8+3665710*X	0.9992	0.04	0.12
Quercetin	8.24	301.026	179.02 (20); 151.01 (22)	Y = -29682.2+1195860*X	0.9987	0.06	0.20
Rutin	6.19	609.197	301.20 (32); 255.17 (54)	Y = -117581+5582020*X	0.9961	0.05	0.18
Isoquercitin	6.44	463.002	301.04 (23); 255.17 (44)	Y = 63068.2+6091263*X	0.9938	0.10	0.32
Astragalin	6.93	447.008	284.03 (29); 255.03 (43)	Y = -83785.1+6078010*X	0.9990	0.04	0.13
Isorhamnetin	9.21	314.989	299.96 (51); 271.20 (21)	Y = -36822.8+1217070*X	0.9958	0.08	0.27
Epideoxyloganic acid	5.97	359.120	197.10 (35)	Y = 45274.4+5439140*X	0.9924	0.11	0.36
Quinic acid	0.92	191.001	173.03 (20), 127.02 (30)	Y = 139364+3458001*X	0.9945	0.11	0.38

t_R – retention time; LOD – limit od detection; LOQ – limit od quantification.

Table S6. Orbitrap-MS^n analysis of phenolics and iridoid glucosides in methanol extracts of *N. nuda in situ* flower and leaf, and *in vitro*.

No	Compound name	t_R, min	Molecular formula, [M–H]⁻	Calculated mass, [M–H]–	Exact mass, [M–H]–	Δ ppm	MS^2 Fragments, (% Base Peak)	MS^3 Fragments, (% Base Peak)	MS^4 Fragments, (% Base Peak)	*in situ* flowers	*in situ* leaves	*in vitro*	* *ref*
	Phenolic acid derivatives												
1	**Gallic acid hexoside 1**	6.02	$C_{13}H_{15}O_{10}^-$	331.06707	331.06709	-0.07	125(29), 167(12), 168(98), 169(33), **313**(100), 314(15)	125(82), 150(65), **151**(100), 165(29), 177(20), 193(69)	95(38), 107(60), **123**(100)	+	+	+	1
2	**Protocatechuic acid**	6.14	$C_7H_5O_4^-$	153.01933	153.01954	-1.33	95(20), 107(18), **109**(100), 110(20), 123(22), 125(31)	65(22), 81(17), **91**(100), 93(8)	NA	+	+	+	2
3	**Gallic acid hexoside 2**	6.34	$C_{13}H_{15}O_{10}^-$	331.06707	331.0671	-0.09	125(26), 167(18), 168(84), 169(29), **313**(100), 314(14)	125(94), 150(86), **151**(100), 165(28), 177(29), 193(56)	95(33), 107(70), 109(7), **123**(100)	+	+	+	1
4	**Vanillic acid**	6.54	$C_8H_7O_4^-$	167.03498	167.0351	-0.73	**69**(100), 121(30), 122(25), 123(91), 137(29), 139(28), 152(32)	NA	NA	+	+	+	2
5	**Dihydroxybenzoic acid hexoside 1**	6.59	$C_{13}H_{15}O_9^-$	315.07216	315.07224	-0.26	109(4), **153**(100)	109(71), **123**(100)	77(6), 79(10), 93(43), **95**(100), 105(40)	+	+	+	1
6	**Dihydroxybenzoic acid hexoside 2**	6.74	$C_{13}H_{15}O_9^-$	315.07216	315.07244	-0.89	109(4), **153**(100)	109(71), **123**(100)	77(6), 79(10), 93(43), **95**(100), 105(40)	+	+	+	1
7	**Caffeic acid hexuronide**	6.9	$C_{15}H_{15}O_{10}^-$	355.06707	355.06738	-0.86	113(14), 135(9), 175(14), **179**(100), 191(12), 209(4)	**135**(100)	79(29), **107**(100)	+	+	-	3
8	**Gentisic acid**	7.04	$C_7H_5O_4^-$	153.01933	153.01961	-1.83	107(3), **109**(100), 110(4), 123(32)	64(31), 65(36), 66(14), **81**(100)	NA	+	+	+	2
9	**Caffeic acid hexoside 1**	7.10	$C_{15}H_{17}O_9^-$	341.08781	341.08802	-0.63	135(6), 161(25), **179**(100)	**135**(100)	91(32), 93(75), **107**(100), 117(73)	+	+	-	1
10	**Caffeoyl tartaric acid**	7.19	$C_{13}H_{11}O_9^-$	311.04086	311.04121	-1.14	**149**(100), 179(35)	59(27), **87**(100), 103(89), 131(43)	NA	+	+	+	3
11	**Aesculin**	7.22	$C_{15}H_{15}O_9^-$	339.07216	339.07255	-1.17	**177**(100), 178(5)	89(5), 105(9), **133**(100), 149(9), 177(3)	**89**(100), 105(22)	+	+	+	4
12	**Dihydroxybenzoic acid hexoside 3**	7.25	$C_{13}H_{15}O_9^-$	315.07216	315.07241	-0.82	109(4), **153**(100)	109(51), 123(4), **135**(100)	**91**(100)	+	+	-	1
13	**Caffeic acid hexoside 2**	7.41	$C_{15}H_{17}O_9^-$	341.08781	341.08808	-0.81	135(8), **179**(100), 180(13), 181(60), 237(8), 251(11), 281(17)	**135**(100)	91(8), **107**(100), 109(7), 117(7)	+	+	+	1
14	**Feruloyl tartaric acid**	8.12	$C_{14}H_{13}O_9^-$	325.05651	325.05693	-1.29	**193**(100)	134(53), **149**(100), 178(68)	**134**(100)	+	+	+	5
15	**Benzoyl tartaric acid**	8.20	$C_{11}H_9O_7^-$	253.03538	253.03556	-0.73	103(4), 113(11), **121**(100), 122(5), 131(3)	**77**(100)	NA	+	+	+	/
16	**Ferulic acid**	9.35	$C_{10}H_9O_4^-$	193.05063	193.05087	-1.21	111(6), 134(99), 147(14), **149**(100), 150(10), 178(57)	**134**(100)	**106**(100), 134(8)	+	+	+	2
17	**Methyl 2-hydroxy-3-(3-hydroxy-4-methoxyphenyl) propanoate**	9.65	$C_{11}H_{13}O_5^-$	225.07685	225.07704	-0.86	135(90), 136(6), 177(11), 179(14), **193**(100), 194(6)	133(13), 134(72), **161**(100), 178(31), 193(5)	117(5), **133**(100), 161(4)	+	+	-	6
18	**Rosmarinic acid**	9.69	$C_{18}H_{15}O_8^-$	359.07724	359.0776	-1.01	133(5), **161**(100), 162(9), 179(23), 197(22), 223(9)	**133**(100)	NA	+	+	+	1
19	**Aesculetin**	9.71	$C_9H_5O_4^-$	177.01933	177.01992	-3.32	113(19), 115(19), 129(22), 132(22), 133(56), 134(27), **135**(100)	91(40), **93**(100), 107(74)	NA	+	+	+	7
20	**Caffeic acid**	9.72	$C_9H_7O_4^-$	179.03498	179.03514	-0.91	134(11), **135**(100), 136(9), 143(4), 149(3), 161(7)	93(24), **107**(100)	NA	+	+	+	2
21	**Syringic acid**	9.78	$C_9H_9O_5^-$	197.04555	197.04583	-1.42	135(44), 153(56), 161(42), 179(13), **197**(100), 343(6)	109(31), 135(9), **153**(100), 179(11)	**135**(100)	+	+	+	1
22	**Nepetoidin B1**	9.79	$C_{17}H_{13}O_6^-$	313.07176	313.07212	-1.16	**161**(100), 237(10), 267(14), 268(10), 269(35), 295(8)	**133**(100)	NA	+	+	+	6
23	**Clinopodic acid A**	10.42	$C_{18}H_{15}O_7^-$	343.08233	343.08292	-1.72	135(7), 145(7), **161**(100), 179(12), 181(5), 197(6)	**133**(100)	89(25), 104(17), **133**(100)	+	+	+	3

No	Compound name	t_R, min	Molecular formula, [M–H]⁻	Calculated mass, [M–H]–	Exact mass, [M–H]–	Δ ppm	MS² Fragments, (% Base Peak)	MS³ Fragments, (% Base Peak)	MS⁴ Fragments, (% Base Peak)	*in situ* flowers	*in situ* leaves	*in vitro*	* *ref*
24	**Methyl rosmarinate**	10.60	$C_{19}H_{17}O_8^-$	373.09289	373.09359	-1.87	**135**(100), 161(67), 175(27), 179(73), 197(35), 311(16), 355(18)	79(11), 93(14), 106(48), **107**(100), 135(33)	NA	+	+	+	8
25	***p*-Hydroxybenzoic acid**	10.89	$C_7H_5O_3^-$	137.02442	137.0247	-2.09	**93**(100), 109(7), 110(3), 138(6)	**65**(100)	NA	+	+	+	2
26	**Salvianolic acid C**	11.56	$C_{26}H_{19}O_{10}^-$	491.09837	491.09892	-1.12	179(10), 267(19), 268(3), **311**(100), 312(6), 341(5)	265(4), 267(20), **283**(100), 284(3), 293(18)	173(88), **174**(100), 254(15), 255(70), 265(22)	+	+	+	9
27	**Ethyl caffeate**	11.61	$C_{11}H_{11}O_4^-$	207.06628	207.06661	-1.60	134(8), 135(21), 161(24), **179**(100), 180(8), 207(3)	**135**(100)	79(17), 91(34), **107**(100), 135(25)	+	+	+	10
28	**Nepetoidin B2**	11.67	$C_{17}H_{13}O_6^-$	313.07176	313.07232	-1.77	**161**(100)	**133**(100)	105(53), 115(9), **133**(100)	+	+	+	8
29	**Methyl salvianolate C1**	13.27	$C_{27}H_{21}O_{10}^-$	505.11402	505.11489	-1.72	179(3), 193(5), 267(26), 268(4), **311**(100), 312(11)	267(22), **283**(100), 284(3), 293(17)	109(11), 173(82), **174**(100), 254(13), 255(71)	+	+	+	11
30	**Methyl salvianolate C2**	13.58	$C_{27}H_{21}O_{10}^-$	505.11402	505.11472	-1.38	193(9), 267(32), **311**(100), 312(14), 343(23), 407(22), 487(14)	267(22), **283**(100), 293(16)	109(12), **173**(100), 174(94), 254(12), 255(59)	+	+	+	11
	Flavonoid glycosides and aglycones												
31	**Luleolin 7-*O*-hexuronide 1**	8.87	$C_{21}H_{17}O_{12}^-$	461.07255	461.07279	-0.51	**285**(100), 286(9)	175(87), 199(85), 213(25), 217(72), **241**(100), 243(61)	**197**(100), 198(83), 199(87), 212(10), 213(52)	+	+	+	3
32	**Apigenin 7-*O*-hexoside**	9.36	$C_{21}H_{19}O_{10}^-$	431.09837	431.1005	-4.93	205(4), 249(7), 268(11), **269**(100), 270(12), 311(3)	149(32), 197(40), 224(33), **225**(100), 227(32), 269(50)	169(23), 181(68), 183(34), 196(39), **197**(100)	+	+	+	1
33	**Apigenin 7-*O*-hexuronide**	9.43	$C_{21}H_{17}O_{11}^-$	445.07764	445.07808	-0.99	175(9), **269**(100), 270(9)	149(36), 151(24), 201(30), **225**(100), 227(21)	169(37), **181**(100), 183(35), 196(24), 197(63)	+	+	+	1
34	**Luleolin 7-*O*-hexuronide 2**	9.59	$C_{21}H_{17}O_{12}^-$	461.07255	461.07296	-0.89	**285**(100), 286(14), 323(16)	175(82), 199(70), 213(27), 217(61), **241**(100), 243(58)	197(71), 198(94), **199**(100), 212(12), 213(49)	+	+	+	3
35	**Luteolin 7-*O*-(acetyl)hexuronide 1**	9.82	$C_{23}H_{19}O_{13}^-$	503.08311	503.08341	-0.59	**285**(100), 286(11)	151(34), 175(76), 199(80), 217(59), **241**(100), 243(55)	**197**(100), 198(80), 199(69), 213(40), 214(13)	+	+	-	12
36	**Luteolin 7-*O*-(acetyl)hexuronide 2**	10.16	$C_{23}H_{19}O_{13}^-$	503.08311	503.08427	-2.30	285(74), 286(12), 309(6), 369(5), **443**(100), 444(21)	284(15), **285**(100), 286(8), 367(6), 399(17)	151(43), 175(83), 199(84), 217(74), **241**(100)	+	+	-	12
37	**Luteolin 7-*O*-(caffeoyl)hexuronide**	10.20	$C_{30}H_{23}O_{15}^-$	623.10424	623.10538	-1.83	245(5), 285(94), 286(11), **337**(100), 338(14), 443(12)	**161**(100), 179(4), 203(5), 219(8), 245(14), 277(5)	**133**(100)	+	+	+	13
38	**Apigenin 7-*O*-hexuronide methyl ester**	10.38	$C_{22}H_{19}O_{11}^-$	459.09329	459.09395	-1.46	**268**(100), 269(45), 270(7), 283(5), 311(5)	151(29), 183(33), 225(100), 240(47), **268**(100), 269(46)	169(31), 181(75), 183(44), 195(12), **197**(100)	+	+	-	14
39	**Apigenin 7-*O*-(caffeoyl)hexuronide**	10.75	$C_{30}H_{23}O_{14}^-$	607.10933	607.11014	-1.34	245(3), **337**(100), 338(11), 427(6)	**161**(100), 203(5), 219(7), 245(13)	**133**(100)	+	+	+	15
40	**Apigenin 7-*O*-(acetyl)hexuronide**	10.80	$C_{23}H_{19}O_{12}^-$	487.0882	487.08879	-1.22	269(22), **427**(100), 428(23)	**268**(100), 269(90), 270(8), 283(7), 311(11), 365(5)	224(29), 225(70), 240(47), **268**(100), 269(41)	+	+	-	/
41	**Luleolin 7-*O*-(feruloyl)hexuronide 1**	10.87	$C_{31}H_{25}O_{15}^-$	637.11989	637.12097	-1.70	285(61), 286(8), 337(13), 351(12), **443**(100), 444(20), 555(13)	284(8), **285**(100), 286(11), 327(5), 367(5), 399(14)	151(49), 175(80), 199(90), 217(77), **241**(100)	+	+	+	/
42	**Luteolin**	10.96	$C_{15}H_9O_6^-$	285.04046	285.04086	-1.40	151(41), 175(98), 199(89), 217(71), **241**(100), 243(59), 285(57)	**197**(100), 198(98), 199(78), 213(48), 226(18), 241(10)	155(12), **169**(100), 170(9), 179(17), 182(35)	+	+	+	1
43	**Luleolin 7-*O*-(feruloyl)hexuronide 2**	11.07	$C_{31}H_{25}O_{15}^-$	637.11989	637.12108	-1.86	**285**(100), 286(12), 409(6), 443(6), 605(14), 606(5), 619(5)	151(40), **175**(100), 199(89), 217(64), 241(96), 243(61)	91(12), 119(79), 131(78), 133(16), **147**(100)	+	+	+	/
44	**Apigenin 7-*O*-(sinapoyl)hexuronide**	11.33	$C_{32}H_{27}O_{15}^-$	651.13554	651.13696	-2.19	205(4), 269(9), 289(5), 381(16), **427**(100), 428(6), 488(4)	**268**(100), 269(59), 283(5), 311(6), 365(4)	224(38), 225(88), 240(60), **268**(100), 269(37)	+	+	+	/

No	Compound name	t_R, min	Molecular formula, [M–H]⁻	Calculated mass, [M–H]–	Exact mass, [M–H]–	Δ ppm	MS[2] Fragments, (% Base Peak)	MS[3] Fragments, (% Base Peak)	MS[4] Fragments, (% Base Peak)	*in situ* flowers	*in situ* leaves	*in vitro*	* *ref*
45	**Apigenin 7-*O*-(feruloyl)hexuronide 1**	11.49	$C_{31}H_{25}O_{14}^{-}$	621.12498	621.12601	-1.66	269(8), 351(11), **427**(100), 428(13)	**268**(100), 269(72), 270(6), 283(4), 311(7), 365(4)	225(69), 239(21), 240(52), **268**(100), 269(36)	+	+	+	/
46	**Apigenin 7-*O*-(feruloyl)hexuronide 2**	11.74	$C_{31}H_{25}O_{14}^{-}$	621.12498	621.12591	-1.50	**269**(100), 270(8), 351(3), 427(4)	149(27), 183(26), 197(32), **225**(100), 227(28), 269(34)	179(9), **181**(100), 183(28), 196(27), 197(76)	+	+	-	/
47	**Apigenin**	11.86	$C_{15}H_{9}O_{5}^{-}$	269.04555	269.04594	-1.47	149(44), 151(27), 183(17), 201(28), **225**(100), 227(17), 269(25)	169(16), 180(19), **181**(100), 183(36), 196(13), 197(35)	71(24), 117(19), **141**(100), 153(15), 180(27)	+	+	+	3
48	**Thymusin**	12.30	$C_{17}H_{13}O_{7}^{-}$	329.06668	329.06714	-1.42	171(12), 291(4), 299(4), 309(8), **314**(100), 315(14)	296(14), **299**(100)	199(9), 227(5), 243(14), 255(25), **271**(100)	+	+	+	3
49	**Apigenin 7-*O*-(acetyl-caffeoyl)hexuronide**	12.42	$C_{32}H_{27}O_{14}^{-}$	635.14063	635.14155	-1.45	268(3), **269**(100), 270(9), 283(6), 441(3), 459(3)	149(36), 183(24), 197(29), 201(24), **225**(100), 269(42)	169(48), 181(55), 183(22), 196(27), **197**(100)	+	+	-	/
50	**Cirsimaritin**	13.34	$C_{17}H_{13}O_{6}^{-}$	313.07176	313.07209	-1.05	283(4), **298**(100), 299(11)	269(25), 270(4), **283**(100)	163(8), 211(5), 227(11), 239(6), **255**(100)	+	+	+	3
51	**Xanthomicrol**	13.65	$C_{18}H_{15}O_{7}^{-}$	343.08233	343.08272	-1.13	297(4), 307(3), **328**(100), 329(17)	**313**(100), 314(4)	284(34), 285(99), 295(12), **298**(100)	+	+	+	1
	Iridoid glycosides												
52	**Nepetanudoside B**	5.98	$C_{16}H_{21}O_{9}^{-}$	357.11911	357.11962	-1.43	213(18), 277(17), 309(83), 310(39), **311**(100), 312(27), 324(15)	101(18), 113(18), 149(42), 159(23), **161**(100), 267(39)	NA	+	+	-	16
53	**Dihydrocornic acid**	6.77	$C_{16}H_{23}O_{10}^{-}$	375.12967	375.12994	-0.71	125(7), 151(64), 152(8), 169(71), 195(15), **213**(100), 214(10)	87(4), 107(9), 125(12), **151**(100), 169(63)	107(58), 109(77), 122(3), 123(6), **133**(100)	+	+	+	17
54	**Secologanin**	7.19	$C_{17}H_{23}O_{10}^{-}$	387.12967	387.1308	-2.91	165(11), 179(11), **225**(100), 226(11), 341(5)	163(5), **165**(100)	109(15), **121**(100)	+	-	+	18
55	**Epideoxyloganic acid hexoside 1**	7.80	$C_{22}H_{33}O_{14}^{-}$	521.18758	521.18815	-1.09	153(51), 154(6), **197**(100), 198(8), 221(5), 359(11), 503(4)	109(28), 135(10), **153**(100)	**135**(100)	+	+	+	8
56	**Nepetanudoside**	7.99	$C_{17}H_{25}O_{10}^{-}$	389.14532	389.14555	-0.60	163(8), 207(11), 209(8), **227**(100), 357(23), 358(3), 371(4)	69(4), **101**(100)	**69**(100)	+	+	+	17
57	**Epideoxyloganic acid pentoside**	8.23	$C_{21}H_{31}O_{13}^{-}$	491.17702	491.17745	-0.89	153(77), **197**(100), 198(9), 233(15), 293(13), 359(18), 445(16)	109(20), 135(6), **153**(100)	**135**(100)	+	+	+	/
58	**Epideoxyloganic acid hexoside 2**	8.26	$C_{22}H_{33}O_{14}^{-}$	521.18758	521.18759	-0.03	153(37), **197**(100), 323(21), 341(28), 359(33), 431(20), 473(14)	109(28), 135(7), **153**(100)	**135**(100)	+	+	+	8
59	**Epideoxyloganic acid 1**	8.32	$C_{16}H_{23}O_{9}^{-}$	359.13476	359.13488	-0.34	109(4), 135(30), 153(67), **197**(100)	109(25), 135(9), **153**(100)	**135**(100)	+	+	+	8
60	**Geniposidic acid**	8.40	$C_{16}H_{21}O_{10}^{-}$	373.11402	373.11403	-0.01	131(9), 149(10), **193**(100), 197(6), 329(43), 330(8), 355(12)	89(44), 101(7), 113(30), 119(9), **131**(100), 149(71)	**113**(100)	+	+	+	19
61	**Epideoxyloganic acid 2**	8.52	$C_{16}H_{23}O_{9}^{-}$	359.13476	359.13485	-0.27	109(5), 135(30), 153(64), **197**(100), 198(4), 313(3)	109(30), 135(8), **153**(100)	**135**(100)	+	+	+	8
62	**Epideoxyloganic acid 3**	9.46	$C_{16}H_{23}O_{9}^{-}$	359.13476	359.13482	-0.18	135(44), 153(56), 161(42), 179(13), **197**(100), 198(13), 343(6)	109(31), 135(9), **153**(100), 179(11)	**135**(100)	+	+	+	8
	Other compounds												
63	**12-*O*-hexosyl-jasmonate**	7.88	$C_{18}H_{27}O_{9}^{-}$	387.16606	387.16607	-0.03	163(80), 164(8), **207**(100), 295(11), 325(13), 369(20)	**163**(100)	81(9), 95(15), 107(21), **109**(100), 147(12)	+	+	+	1

*1 – Abu-Reidah et al. (2019); 2 – Kaska et al. (2018); 3 – Кащенко and Николаевич (2016); 4 – Mišić et al. (2015); 5 – Carocho et al. (2015); 6 – Fraga et al. (2017); 7 – Wu et al. (2020); 8 – Aničić et al. (2021); 9 – Taamalli et al. (2015); 10 –Saltos et al. (2014); 11 – Ma et al. (2011); 12 – Ferreira et al. (2012); 13 – Moghadam et al. (2015); 14 – Lehbili et al. (2018); 15 – Lee et al. (2003); 16 – Guvenalp et al. (2009); 17 – Goldansaz et al. (2019); 18 – Pan et al. (2020); 19 – Frezza et al. (2019).

References

Abu-Reidah, I.M., Arráez-Román, D., Al-Nuri, M., Warad, I., Segura-Carretero, A. (2019). Untargeted metabolite profiling and phytochemical analysis of *Micromeria fruticosa* L. (Lamiaceae) leaves. *Food Chem*. 279, 128–143. doi: 10.1016/j.foodchem.2018.11.144

Aničić, N., Gašić, U., Lu, F., Ćirić, A., Ivanov, M., Jevtić, B., et al. (2021). Antimicrobial and Immunomodulating Activities of Two Endemic *Nepeta* Species and Their Major Iridoids Isolated from Natural Sources. *Pharmaceuticals (Basel)* 14:414. doi: 10.3390/ph14050414

Carocho, M., Barros, L., Calhelha, R. C., Ćirić, A., Soković, M., Santos-Buelga, et al. (2015). *Melissa officinalis* L. decoctions as functional beverages: a bioactive approach and chemical characterization. *Food Funct.* 6, 2240– 2248. doi: 10.1039/c5fo00309a

Cuenoud, P., Savolainen, V., Chatrou, L.W., Powell, M., Grayer, R.J., and Chase, M.W. (2002). Molecular phylogenetics of Caryophyllales based on nuclear 18S rDNA and plastid rbcL, atpB, and matK DNA sequences. *Am. J. Bot.* 89, 132–144. doi: 10.3732/ajb.89.1.132

Ferreira, F.M., Dinis, L.T., Azedo, P., Galhano, C.I.C., Simões, A. Cardoso, S.M., et al. (2012). Antioxidant capacity and toxicological evaluation of *Pterospartum tridentatum* flower extracts. *CyTA--J. Food* 10, 92–102. doi: 10.1080/19476337.2011.590233

Fraga, B.M., González-Coloma, A., Alegre-Gómez, S., López-Rodríguez, M., Amador, L.J., Díaz, C. E. (2017). Bioactive constituents from transformed root cultures of *Nepeta teydea*. *Phytochemistry* 133, 59–68. doi: 10.1016/j.phytochem.2016.10.008

Frezza, C., Venditti, A., Giuliani, C., Foddai, S., Maggi, F., Fico, G., et al. (2019). Preliminary study on the phytochemical evolution of different Lamiaceae species based on iridoids. *Biochem. Syst. Ecol.* 82, 44–51. doi: 19. 10.1016/j.bse.2018.12.003

Goldansaz, S. M., Festa, C., Pagano, E., De Marino, S., Finamore, C., Parisi, O. A., et al. (2019). Phytochemical and Biological Studies of *Nepeta asterotricha* Rech. f. (Lamiaceae): Isolation of Nepetamoside. *Molecules* 24:1684. doi: 10.3390/molecules24091684

Guvenalp, Z., Özbek, H., Kuruuzum-Uz, A., Kazaz, C., Demirezer, L.Ö. (2009). Secondary metabolites from *Nepeta heliotropifolia*. *Turk. J. Chem.* 33, 667–675. doi: 10.3906/kim-0812-60

Kaska, A., Deniz, N., Çiçek, M., Mammadov, R. (2018). Evaluation of Antioxidant Properties, Phenolic Compounds, Anthelmintic, and Cytotoxic Activities of Various Extracts Isolated from *Nepeta cadmea*: An Endemic Plant for Turkey. *J. Food Sci.* 83, 1552–1559. doi: 10.1111/1750-3841.14167

Lee, J.S., Kim, H.J., Lee, Y.S. (2003). A new anti-HIV flavonoid glucuronide from *Chrysanthemum morifolium*. *Planta Med.* 69, 859–861. doi: 10.1055/s-2003-43207

Lehbili, M., Alabdul Magid, A., Kabouche, A., Voutquenne-Nazabadioko, L., Abedini, A., Morjani, H., et al. (2018). Antibacterial, antioxidant and cytotoxic activities of triterpenes and flavonoids from the aerial parts of *Salvia barrelieri* Etl. *Nat. Prod. Res.* 32, 2683–2691. doi: 10.1080/14786419.2017.1378207

Levin, R.A., Wagner, W.L., Hoch, P.C., Nepokroeff, M., Pires, J.C., Zimmer, E.A., et al. (2003). Family-Level Relationships of Onagraceae Based on Chloroplast *rbcL* and *ndhF* Data. *Am. J. Bot.* 90, 107–115. doi: 10.3732/ajb.90.1.107

Ma, H.Y., Gao, H.Y., Sun, L., Huang, J., Xu, X.M., Wu, L.J. (2011). Constituents with α-glucosidase and advanced glycation end-product formation inhibitory activities from *Salvia miltiorrhiza* Bge. *J. Nat. Med.* 65, 37–42. doi: 10.1007/s11418-010-0453-2

Mišić, D., Šiler, B., Gašić, U., Avramov, S., Živković, S., Nestorović Živković, J., et al. (2015). Simultaneous UHPLC/DAD/(+/–)HESI–MS/MS Analysis of Phenolic Acids and Nepetalactones in Methanol Extracts of *Nepeta* Species: A Possible Application in Chemotaxonomic Studies. *Phytochem. Anal.* 26, 72–85. doi: 10.1002/pca.2538

Moghadam, S.E., Ebrahimi, S.N., Gafner, F., Ochola, J.B., Marubu, R.M., Lwande, W., et al. (2015). Metabolite profiling for caffeic acid oligomers in *Satureja biflora*. *Ind. Crops Prod.* 76, 892–899. doi: 10.1016/j.indcrop.2015.07.059

Pan, H., Zhou, H., Miao, S., Cao, J., Liu, J., Lan, L., et al. (2020). An integrated approach for global profiling of multi-type constituents: Comprehensive chemical characterization of *Lonicerae Japonicae* Flos as a case study. *J. Chromatogr. A* 1613: 460674. doi: 10.1016/j.chroma.2019.460674

Saltos, M.B., Puente, B.F., Malafronte, N., Braca, A. (2014). Phenolic compounds from *clinopodium tomentosum* (Kunth) govaerts (Lamiaceae). *J. Braz. Chem. Soc.* 25, 2121–2124. doi: 10.5935/0103-5053.20140181

Sang, T., Crawford, D.J., and Stuessy, T.F. (1997). Chloroplast DNA phylogeny, reticulate evolution, and biogeography of *Paeonia* (Paeoniaceae). *Am. J. Bot.* 84, 1120–1136.

Stanford, A.M., Harden, R., and Parks, C.R. (2000). Phylogeny and biogeography of *Juglans* (Juglandaceae) based on matK and ITS sequence data. *Am. J. Bot.* 87, 872–882.

Taamalli, A., Arráez-Román, D., Abaza, L., Iswaldi, I., Fernández-Gutiérrez, A., Zarrouk, M., et al. (2015). LC-MS-based metabolite profiling of methanolic extracts from the medicinal and aromatic species *Mentha pulegium* and *Origanum majorana*. *Phytochem. Anal.* 26, 320–330. doi: 10.1002/pca.2566

Tate, J.A., and Simpson, B.B. (2003). Paraphyly of *Tarasa* (Malvaceae) and diverse origins of the polyploid species. *Syst. Bot.* 28, 723–737. doi: 10.1043/02-64.1

Valyova, M.S., Dimitrova, M.A., Ganeva, Y.A., Kapchina-Toteva, V., and Yordanova, Zh.P. (2011). Evaluation of antioxidant and free radical scavenging potential of *Lamium album* L. growing in Bulgaria. *J. Pharm. Res.* 4, 945-947.

White, T.J., Bruns, T., Lee, S., and Taylor, J. (1990). "Amplification and direct sequencing of fungal ribosomal RNA genes for phylogenetics.", in PCR Protocols: a guide to methods and applications, eds. M. A. Innis, D. H. Gelfand, J. J. Sninsky, T. J. White (Academic Press, New York, USA), 315–322.

Wu, C., Liu, H., Rong, X., Liu, J., Ding, W., Cheng, X., Xing, J., Wang, C. (2020). Phytochemical composition profile and space-time accumulation of secondary metabolites for *Dracocephalum moldavica* Linn. via UPLC-Q/TOF-MS and HPLC-DAD method. *Biomed. Chromatogr*. 34:e4865. doi :10.1002/bmc.4865

Кащенко (Kashchenko), Н. (Nina) И. (Igorevna) and Оленников (Olennikov), Д. (Daniil) Н. (Nikolaevich) (2016). Химический профиль и биологическая активность флавоноидов и фенилпропаноидов *Nepeta Cataria* L. (Lamiaceae), интродуцированного в восточной Сибири (Chemical Profile and Biologocal Actitity of Flavonoids and Phenylpropanoids from *Nepeta Cataria* L. (Lamiaceae) Cultivated in the Eastern Siberia). *Химия растительного сырья* 2, 25–32. doi: 10.14258/jcprm.2016021084
